# Institutionalization and home health care after acute hospitalizations of older persons in Norway

**DOI:** 10.1186/s12913-025-13851-x

**Published:** 2025-12-17

**Authors:** Katrine Damgaard Skyrud, Siri Rostoft, Astri Syse

**Affiliations:** 1 Cluster for Health Services Research, Norwegian Institute of Public Health, Oslo, Norway; 2 Department of Geriatric Medicine, Oslo University Hospital, Oslo, Norway; 3 Institute of Clinical Medicine, Faculty of Medicine, University of Oslo, Oslo, Norway

**Keywords:** Ageing, Care, Formal, Health, Long-term care, Services, Sociodemographic, Trauma

## Abstract

**Background:**

Population ageing and strained public resources challenge the future provision of health and care services. We assess the uptake of formal health-related care services (FCS), either home health care (HHC) or short- or long-term institutional care (IC), before and after an acute hospital admission in a full-population sample of older persons.

**Methods:**

Multinominal logistic population average regression models were applied to Norwegian registry data on older persons (aged 75+) in 2021 (*N* = 68,803) at hospital discharge following admission for select diagnoses (*N* = 94,748) to examine how functional status (for FCS users) and comorbidities (for non-FCS users), acute hospital admissions and sociodemographic characteristics are associated with HHC and IC uptake within 4 weeks and at 6 months post-discharge.

**Results:**

Altogether, 53% of the sample were already recipients of FCS at the time of the acute event, 43% in HHC and 10% in IC. Overall, the shares increased to 33% for IC and decreased to 29% for HHC 4 weeks post-discharge. At 6 months, the respective shares were 14 and 35%. Among HHC recipients at hospital admission, 44% transitioned to IC within 4 weeks. At 6 months, the share had declined to 21%. Multivariate models showed that transitions into and within FCS were associated with older age, female sex, comorbidities/functional status and living alone. Trauma, cerebrovascular and geriatric conditions were substantially associated with subsequent FCS uptake, and especially IC. Short-term IC transitions were more common among HHC-users than non-HHC-users. In terms of moderating factors, larger differences were observed by living situation as opposed to sex and functional status/comorbidity.

**Conclusions:**

Irrespective of prior FCS uptake, acute hospital admissions are associated with subsequent FCS uptake, especially for trauma, cerebrovascular and geriatric conditions. Minor variations across sociodemographic characteristics suggest need-based, equitable service provision. The mode (HHC vs IC) and temporality of FCS uptake warrants further research to identify policy measures that may improve care trajectories to ensure sustainable, safe, and high-quality care and rehabilitation following acute hospitalizations of older individuals. Clinical practice and future research should include hospital frailty measures to improve predictions of future FCS needs, particularly for persons unknown to the FCS system pre-hospitalization.

**Supplementary Information:**

The online version contains supplementary material available at 10.1186/s12913-025-13851-x.

## Background

With widespread population ageing, many Western countries can expect a substantial growth in demand for labour-intensive health and care services, provided both at hospitals and within the formal care services (FCS^1^) [1, 2]. Although prior research suggests that prevention efforts, including primary, secondary, and tertiary preventive measures to some extent can reduce the need for acute hospital admissions [3], older adults admitted acutely occupy a majority of emergency hospital beds in many Western countries [4]. An important aim of effective acute hospital care for older people is thus to assess individual needs, treat modifiable conditions, support functional recovery, and facilitate a rapid discharge, with or without FCS [4, 5]. Knowledge of how patients move between the various systems of health and care is important to ensure high-quality treatment and avoid fragmented health and care trajectories, but there is scarce knowledge of subsequent short- and long-term FCS uptake either at home or in institutions in population-based samples of older individuals after acute hospital admissions.

Older adults are more likely to have various comorbidities and disabilities than younger adults, and typically require a longer time to recover from acute illness [3]. Acute hospital admissions among older persons are thus often accompanied by temporary or permanent functional decline [3, 6], physical dependence, and (increased) needs for FCS [3, 6, 7]. Typical risk factors associated with functional decline among hospitalized older patients include older age, preexisting disability and/or functional status, including cognitive impairment upon admission, along with type of illness or injury, and aspects of the hospital stay itself [3, 6]. The acute event, such as a stroke, a hip fracture, or a serious infection, may adversely alter functional status, permanently or temporarily, and thus impact on post-discharge FCS needs. The hospitalization episode may also result in deconditioning (e.g., limited mobility/bedrest, side effects of medical procedures such as diagnostic tests and polypharmacy) that may be accompanied by a functional loss and/or reduced independence. Lastly, institutionalization and associated processes may promote continued institutionalized care [6].

Home health care (HHC) is the most commonly awarded FCS among older individuals in Western countries [1] and is the preferred form for care, when appropriate [1], also in Norway [8]. More than 80% of all FCS users in Norway are HHC recipients, and whereas 27% of individuals aged 80 or above use HHC, 11% rely on institutionalized care (IC) [9]. The shares are similar in many other OECD countries, although the IC figures are generally lower in the US [2]. Following hospitalization, the share relying on post-acute care is, however, relatively high (40%) also in the US setting, and also here the majority is awarded HHC (rather than IC) [2]. As HHC and IC may substitute one another, it is valuable to take a joint perspective across both care forms.^2^

### Existing research

Much of the research on short- and long-term consequences of acute hospitalizations of older individuals examines hospital readmissions [10, 11]^3^, and there is less focus on transfers to IC and HHC. Existing literature shows great variation in post-hospital discharge trajectories and uptake of FCS [12], but there is more focus on transfers to and/or uptake of IC [12] rather than HHC [13]. Furthermore, studies that *do* examine IC transfers, often fail to include HHC uptake as a possible substitute, and vice versa. Below we provide examples of studies that examine either IC or HHC uptake following acute hospitalization of older persons, before we summarize the few studies that have examined these care forms jointly.

### Institutionalized care

IC admission may be necessary and offer the best care after discharge from acute hospitalization, and knowledge of IC predictors is critical to inform effective care service planning [14]. IC may also reduce the demand on hospitals if acute events can be handled appropriately within the IC setting [13]. A systematic review from 2017 found shares of hospital discharges to IC to vary from 3 to 77% [12], partly attributed to differences across types of facilities, contexts and study quality. In addition, the use of (various types of) IC has changed over time [6, 15]. One the one hand, an older US study examining risk of institutionalization 6 months post-hospitalization found that the risk of long-term IC was quite low (6%) and declined markedly from the late 1990s to 2008 for older patients admitted to hospital from home [6]. Nevertheless, around three quarters of new IC placements were precipitated by a hospitalization [6]. On the other hand, more recent US studies show that the number of patients discharged to post-acute care facilities after hospitalization is relatively high. According to one study, this pertained to 25% of the 65 + and 40% of the 80 + year olds, and the shares increased by 50% in the same time period [15]. Thus, different results are observed for various forms of IC, perhaps due to shortened hospital stays, more post-acute care facilities, advanced HHC, and a growth of other community-based alternatives such as assisted living facilities, in line with what is observed in many European countries [16].

Previous work has identified important associations between social care receipt in general and older age, cognitive and functional impairment as well as multimorbidity, requirement for support in activities of daily living (ADL), not owning your home, polypharmacy, area-based deprivation and living alone [7, 17, 18]. The latter point is also underscored in a Canadian study, where social vulnerability was found to be associated with increased risk of IC placement [19]. However, there was no difference along this dimension among those who were frailer, suggesting that at a certain age and frailty level, such placements may be difficult to avoid even within supportive social situations [19]. This is also seen in a study examining pathways into IC from hospitals versus communities, which found that individuals moving into IC directly from hospitals had greater dependency, frailty and recent health events that included fractures, strokes, and significant mental illnesses [5]. The risk factors associated with IC placement after acute hospitalizations appear similar to the general risk factors for IC uptake, and include functional dependency, comorbidities and/or polypharmacy, cognitive impairment and/or dementia, female sex and older age [4, 12], although not all studies identify sex as an important predictor [7]. Sociodemographic characteristics *other* than age and sex also play a role, such as ethnicity, material deprivation and/or income, living situation and/or marital status, education, and social factors. According to a US study, 10% of older individuals experienced a new IC placement 6 months after hospitalization, and identified white ethnicity, being unmarried, Medicaid eligibility and higher income as important predictors, net of physical and cognitive impairment, but with more pronounced impact of these covariates for the cognitively intact [20]. Lack of social support or caregiver availability measured directly or proxied by living situation and/or marital status is consistently associated with IC placement [3]. In summary, the strengths of associations between risk factors (other than age) and IC transitions across many contexts seem to diminish as age increases, especially if cognition is severely reduced [14, 20, 21].

Whereas there is an increasing trend towards short-term IC stays in newly admitted hospital patients in the US [21] and thus a lower level of direct long-term IC admissions, the level is higher in the UK [14]. Predictors for admission are, however, similar in both settings, and include older age, living alone and functional status, the latter including cognitive issues, neurological symptoms of cerebrovascular disease, fractures, falls and reduced mobility.

The nature of the acute event itself also has an impact, including hospital admission diagnosis and length of hospital stays [14]. The presence of geriatric syndromes at hospital admission has been found to be associated with post-discharge institutionalization or a change in residential care status to a more dependent category [22]. Predictive factors include impaired pre-admission ADL status, recurrent falls, urinary incontinence and supported living arrangements. Falls often result in trauma, irrespective of the presence of geriatric syndromes, and trauma is associated with IC admissions following hospital stays. According to a Norwegian study, 16% of hip fracture patients admitted from home received IC at 6 months [23], and lower ADL scores and delirium, a frequent complication among older hospitalized patients, comprised the main risk factors for such transfers. Among those who *did* return home, half did not return to their pre-fracture level of ADL functionality within 6 months [23].

In summary, discharges to (long-term) IC after acute hospitalizations are common, but marked variations exist in IC set-up and financiering, frequency of use, and hospital and patient characteristics that have an impact across contexts [12].

### Home health care

Recovering at home after discharge from an acute hospitalization is often aligned with patients’ preferences [6, 20], avoids some of the known risks associated with hospitalization and IC [4], and lowers health care spending [24]. Returning to and remaining at home is thus a highly desirable outcome for patients, family carers, the health services and society in general [4], but requires high-quality and safe HHC. The knowledge base is nevertheless more limited for HHC than for IC after an acute hospitalization [13]. Existing studies show that HHC (vs. no care) after a hospital admission was associated with both lower mortality and fewer hospital readmissions, suggesting that HHC after hospitalization may improve continuity of care and improve patient outcomes [25–28].

The threshold for HHC appears to vary markedly across contexts. A Norwegian study shows that older patients who receive HHC are characterized by low life expectancies, multiple hospital admissions and the majority show signs of cognitive impairment [29]. According to a UK study, 9 in 10 older adults achieve the positive outcome of living at home 4 weeks after discharge [4]. Older patients and patients with dementia and delirium had the greatest risk of not living at home [4]. Patient sex, area-based deprivation and polypharmacy did not matter [4]. A US study estimated time trends in the frequency and duration of HHC among older adults discharged home after a hospital stay and found that 44% reported receiving help within 3 months of discharge [24]. Compared with people not receiving help, those receiving help were older, had worse health and were more likely to have dementia. The percentage of respondents who reported receiving help post-hospitalization increased markedly from 2011 (38%) to 2017 (52%). For those who were independent in their ADLs before hospitalization, the share receiving help after discharge increased even more. Among those who received help after discharge, the need for help slowly declined to prehospitalization levels over the ensuing 9 months.

Few studies have specifically examined outcomes among individuals discharged back into the community [30]. Those that do exist, find that receiving HHC services is associated with better clinical outcomes [27, 30]. A comparison of outcomes between hospitalized patients discharged home to self-care versus HHC in the US found that HHC was associated with fewer readmissions and lower health care cost over the first year after hospitalization, although the HHC lasted for 3 months or more [27]. The cost savings associated with fewer hospital readmissions thus offset parts of the cost of HHC for the population who received HHC, which is also underscored in a recent review [31]. However, although 1 in 3 US Medicare HHC episodes are preceded by a stay at a hospital, skilled nursing facility, or inpatient rehabilitation facility [28], differential barriers for HHC access have been suggested as only half of hospital discharges with a home health referral actually *received* HHC within 14 days of discharge and the sociodemographic disadvantaged individuals were the least likely to receive such care [32].

In summary, transitions to HHC among older adults discharge from hospital appear to be a complex process for both the health care system, patients and their formal and informal caregivers [33].

### Comparing home health and institutionalized care

Studies show that compared with patients discharged home (with or without HHC), patients discharged to IC are older, have longer hospital stays, require more ADL support, and have more comorbidities, including cognitive impairment [15, 34]. The importance of multimorbidity is also observed in a Scottish study, where the vast majority (93%) of those receiving social care had multimorbidity [17]. Turning the issue around, it was observed that whereas 16% of those with multimorbidity received social care, the number was 4% among those without [17]. Deprivation increased the risk for social care uptake [17]. That cognitive issues are more severe in those admitted to IC versus HHC, is also found in other studies [4, 29]. A UK study examining chances of living at home after an emergency hospital admission in older people with and without cognitive problems found, for instance, that those without cognitive problems were more likely to live at home after 4 weeks and much less likely to receive IC, whereas cognitively impaired persons were at significantly increased risk of not returning home after hospitalisation and much more likely to be admitted to IC [4].

An older study of factors associated with 3 months discharge destinations for older persons acutely admitted to hospitals in a comparative European study, found that whereas the majority (85%) had returned to their respective homes, around 6% received IC (the remaining 9% had died) [35]. Physical functioning was the most important predictor [35], and in contrast to more recent work [4], cognitive functioning did not emerge as an important predictor. Otherwise, the findings were consistent in the two studies, in that the majority were discharged home to their previous residence, with predictors of institutional care identified as physical function, living alone, presence of ‘geriatric giants’ on admission, age and sex [4, 35].

A US study comparing outcomes between hospitalized patients discharged to IC (61%) versus HHC (39%) found that HHC was associated with higher hospital readmission rates, but that no differences were observed in functional status [36]. Medicare spending was, however, lower for those discharged to HHC in the first- and second-month post-discharge. A US study examining older adults discharged from hospital to a skilled nursing facility and then home, found that while two-thirds of Medicare beneficiaries admitted to HHC after a stay at IC recovered functionally by 3 months, patients who were frail experienced an overall longer duration of HHC services (>3 months) due to prolonged functional ADL dependence [30]. According to the same study, recovery decreased after 2 months, possibly reflecting continued high care needs.

To summarize, nearly half of hospitalized older adults are not discharged home (with or without HHC) immediately, but are re-hospitalized, die, or move into IC [20, 37]. Of older adults discharged to HHC, many still develop new ADL disabilities compared with their premorbid status. Furthermore, two-thirds of individuals discharged to (short-term) IC are later admitted to HHC services [30]. Thus, understanding the expected length of recovery and the care trajectory is critical for care coordination and long-term planning for patients and families, who may not expect the intensity and duration of support a recovering older adult requires, either in IC or at home (with or without HHC). The decline in use of post-acute IC mean that more patients are going directly home after hospital discharge, and the consequences are largely unknown in terms of extent, quality, and safety.

### Context, aims and research questions

Norway sits within the Nordic tradition of extensive and universal welfare provision of both health and care services [38]. Norway’s National Insurance Scheme ensures highly subsidized healthcare and FCS (either home-based or institutionalized) to all residents regardless of age [38]. Whereas the state is responsible for the provision of hospital services, the delivery of FCS is the responsibility of Norwegian municipalities. A hospital discharge thus implies that the care responsibility is carried over to a different governmental level, with hospital personnel having little or no say in the level of further care pathways once the patient is considered medically stable enough to be discharged from specialized health care. In Norway, FCS are predominantly publicly financed (85%) through general taxation. FCS are rationed according to needs, which should make access to FCS relatively independent from patients’ economic or familial resources [38]. Still, recent research indicates fewer IC transitions among resourceful older individuals, in contrast to those who are less resourceful [39]. Individual resources beyond age and sex, such as living situation, educational level and income thus appear to modify the need for FCS, net of established risk factors (preexisting disability and/or clinical status, cognitive impairment upon admission, depressive symptoms, and delirium, type of illness or injury, as well as aspects of the hospital stay itself). Relevant mechanisms could be health literacy or greater sources of informal care and support. To our knowledge, no prior research has been performed to determine how patterns and trajectories might vary following different acute events from a full population perspective, including patients admitted from home with or without HHC (HHC) or from institutionalized care (IC). In this study, we take advantage of Norwegian full-population longitudinal data to examine the two types of post-acute care jointly in a short- (4 weeks) and long-term (6 months) perspective, while also including persons already in receipt of FCS, thereby adding to the existing literature. Given the limited body of existing research, we pose the following three broad research questions (RQs):

#### RQ_1_

What is the likelihood of transitioning into either IC or HHC after an acute hospital admission among older patients?

#### RQ_2_

Is the acute event associated with an increase in the need for IC for patients already in receipt of HHC prior to the acute event?

#### RQ_3_

Does the likelihood of transitioning into either IC or HHC differ by the nature of the acute event, functional status/comorbidity and/or patient characteristics?

### Data and methods

#### Data

We obtained data on health and care utilization from two full-population national registers: the Norwegian Patient Registry (NPR) and the Municipal Patient and User Registry (KPR). Sociodemographic information was obtained from Statistics Norway, and information from January 2021 were used to ensure the characteristics were assessed *prior* to the acute event. The files were linked by unique ID numbers, assigned to all residents in Norway. The NPR contains information on all admissions (outpatient clinic consultation, day and overnight) to Norwegian public hospitals, but we restrict our sample to only include non-elective day or overnight stays (i.e., acute admission). Admission and discharge dates, diagnoses associated with the admission, as well as procedure codes are registered. The KPR contains individual-level information on persons receiving long-term care, ranging from in-home safety alarms to full-time institutionalized care, additionally also physical and cognitive functioning. The most frequently used services include home health care (HHC), practical assistance and institutionalized care (IC), but as we only include assessments of health-related FCS uptake, we limit our analyses to HHC and IC. The sociodemographic information includes month and year of birth, death, and emigration, sex, education, income and living situation.

We collapsed hospital admissions with less than two days between admission and readmission into a single hospitalization stay, as they might be associated with suboptimal care factors and not necessarily represent new medical incidences in older patients.^4^ The data cover 68,803 Norwegian residents aged 75 and older admitted to a public hospital for select diagnostic groups between January 1 and December 31, 2021, responsible for a total of 94,748 hospital admissions. Hospital data from both 2020 and 2021 (one year prior to the event) were used to generate dummies for previous admissions for similar conditions, as well as comorbidity scores for persons not already in receipt of FCS. In the same manner, we used FCS data from 2021 to assess functional status for persons already in receipt of FCS at hospital admission. All data handling has been done on a de-identified file and undertaken in accordance with the Declaration of Helsinki and in line with national ethics requirements.

### Outcome variables

The first outcome we study is *transitions into FCS*, which includes transitions to home nursing and other forms of healthcare (e.g., physiotherapy, occupational therapy, and (re)habilitation services) received in the patient’s home (HHC) or institutionalized care (IC). This applies to persons admitted from home, with no FCS use at time of hospital admission and is measured 0–4 weeks and 6 months after hospital discharge from an acute event. The second outcome we study comprise *transitions within FCS*. This applies to persons admitted from home, with HHC at time of hospital admission. We examine the transition from HHC to IC, in the same time periods described above. The time period 0–4 weeks was chosen to account for the ’acute’ post-discharge phase – in Norway short term stays in IC are planned for a maximum of 3–4 weeks. Stays longer than this typically indicate a permanent need for IC, and therefore we decided to examine the situation also at 6 months post-discharge.^5^

**Fig. 1:**
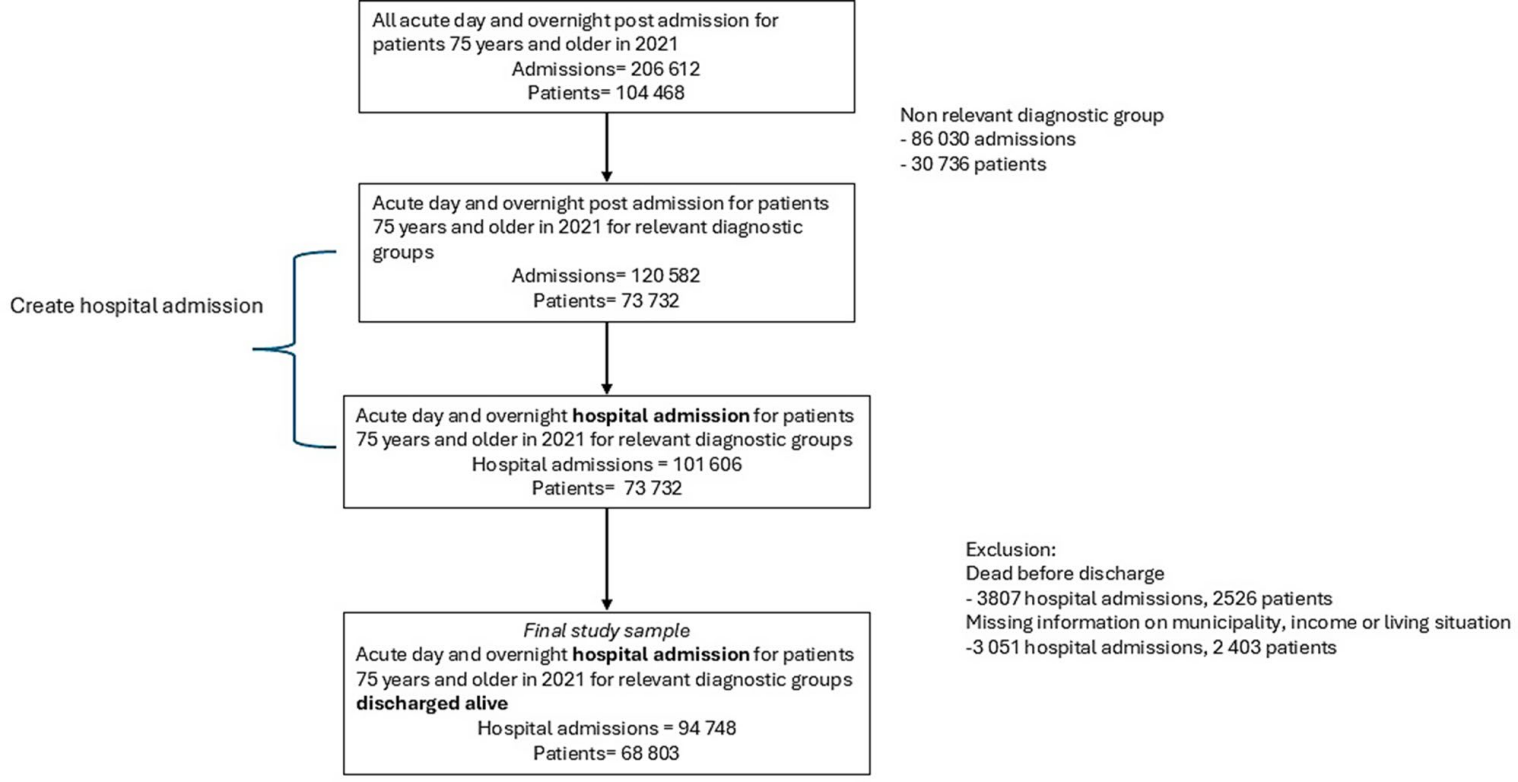
Flowchart of the study population

Persons are censored if death, emigration, or re-hospitalization (at 6 months) occurs, but as these events are relevant for policy planning, a descriptive summary of the extent of censorship is provided. A flowchart of the sample selection is shown in Fig. 1.

### Covariates

We selected the following *acute events*: Infections, heart disease, cerebrovascular disease, trauma, geriatric diseases, along with a control group for which we expect acute events to minimally influence subsequent FCS uptake. The control group was chosen to have a comparison of individuals with diagnoses that would normally not influence a patient’s functional status/care need, such as hypertension, chest pain, transient ischemic attack, dyspepsia and constipation (see Supplement Table S1 for ICD-10 codes). The categorization of the diagnostic groups resulted from consultations with geriatric expertise, based on events that are common in the population of acutely hospitalized older adults. Geriatric disease is a proxy for geriatric syndromes (or ‘geriatric giants’) – acute events common in patients living with frailty, such as falls, delirium, electrolyte disturbances, dehydration, and dementia.

The hospital acute episode was defined as an acute day or overnight stay with one of the select ICD-10 diagnostic codes, based on the last principal diagnosis received at discharge.^6^ Each hospital admission thus corresponds to only one hospital episode. A patient could, however, have more than one event during the study period, and a dummy variable (*prior admission*) indicating that the patient had a prior acute admission with the same diagnostic group in the previous 6 months was included as a control variable in the regression model. The length of hospital stay was trunked at 10 days and included in the model as a linear variable.

*Functional status (*for HHC-users) was measured in relation to ADL independence and grouped into (1) Poor; and (2) Impaired.^7^*Comorbidity* (for those with no prior FCS) was based on the Charlson Comorbidity Index (CCI), based on diagnostic codes for any hospital admission in the prior year [40], categorized into (1) comorbidity (CCI >1); and (2) no comorbidity. *Educational attainment* was dichotomized into (1) Low (compulsory and secondary education); and (2) High (tertiary education), in line with the ISCED-2011 classification.^8^*Household income* was dichotomized into (1) Low income, based on the EU-60 scale^9^; and (2) High income. *Living situation* was categorized as (1) Living alone; and (2) Not living alone. In addition, we group *age* into four groups: 75–79, 80–84, 85–89 and 90 + years and *sex* into two groups: male and female.

### Methods

To assess variations in FCS transitions, we employ multinomial or binary logit population average models (using the generalized estimating equation) with standard errors clustered at the hospital level. To determine if there are variations in FCS transitions according to the nature of the acute event, our initial models include the broad disease groups and adjusts for patient characteristics. Next, we include interaction terms between the broad disease groups and the patient variables, to identify whether the general associations between the nature of the acute event and FCS transitions are moderated by sociodemographic characteristics.

To model the first outcome (transitions into FCS), we used a multinomial logistic regression model, were Y = 2 if IC was received by an individual at any time during the 4-weeks or at 6 months after discharge, Y = 1 if HHC was received by an individual at any point during the 4-weeks or at 6 months after discharge, Y = 0 if no FCS was received during the study period (reference category). In cases where an individual received both type of FCS, the highest level of care was chosen (IC > HHC). The second outcome (transitions within FCS) was modelled with a binary logistics regression, were Y = 1 if IC was received by an individual at any time during the 4- weeks or at 6 months after discharge, and Y = 0 otherwise (reference category).

We applied three different models for the different outcomes, and the models build on one another (nested models). **Model 1** is the simplest model, including only age, sex and functional status/comorbidities. **Model 2** also accounts for factors associated with hospital stay, including diagnostic groups, previous admissions within 6 months for the same diagnostic group, and length of hospital stay. **Model 3** is the most complex model, including also the patient sociodemographic characteristics (income, education and living situation). Formal comparison tests (likelihood ratio) were used to confirm our choice of the final model. Finally, to answer **RQ**_**3**,_ does the likelihood of transitioning into either IC or HHC differ by the nature of the acute event, functional status/comorbidity and/or patient characteristics, we estimated a full model (Model 3) plus interaction term between diagnostic group and the selected patient characteristics (sex, comorbidity/functional status and living situation).

We performed two additional robustness checks to assess the stability of our result. In the first, we included hospital fixed effects in the regression models to control for hospital-level confounding (i.e., remove all variation between hospitals, such as such as hospital size and staffing levels). We only include this as a robustness check since our aim is to describe the overall resource needs associated with acute hospitalizations, irrespectively of the specific hospital affiliations. In the second, we excluded discharges with another hospitalization within 30 days before the index hospitalization for patients who were already receiving HHC prior to the acute event. This was done to account for the potential bias that the likelihood of transitioning into IC may differ between those that need IC because of long-term support and those that need it for post-acute care.

To enable cross-model comparisons, we present marginal effects [41] in the form of predicted probabilities at the mean (all independent variables are set to their mean values). All statistical analysis were performed in R version 4.3.2.

## Results

### Descriptive results

**Fig. 2:**
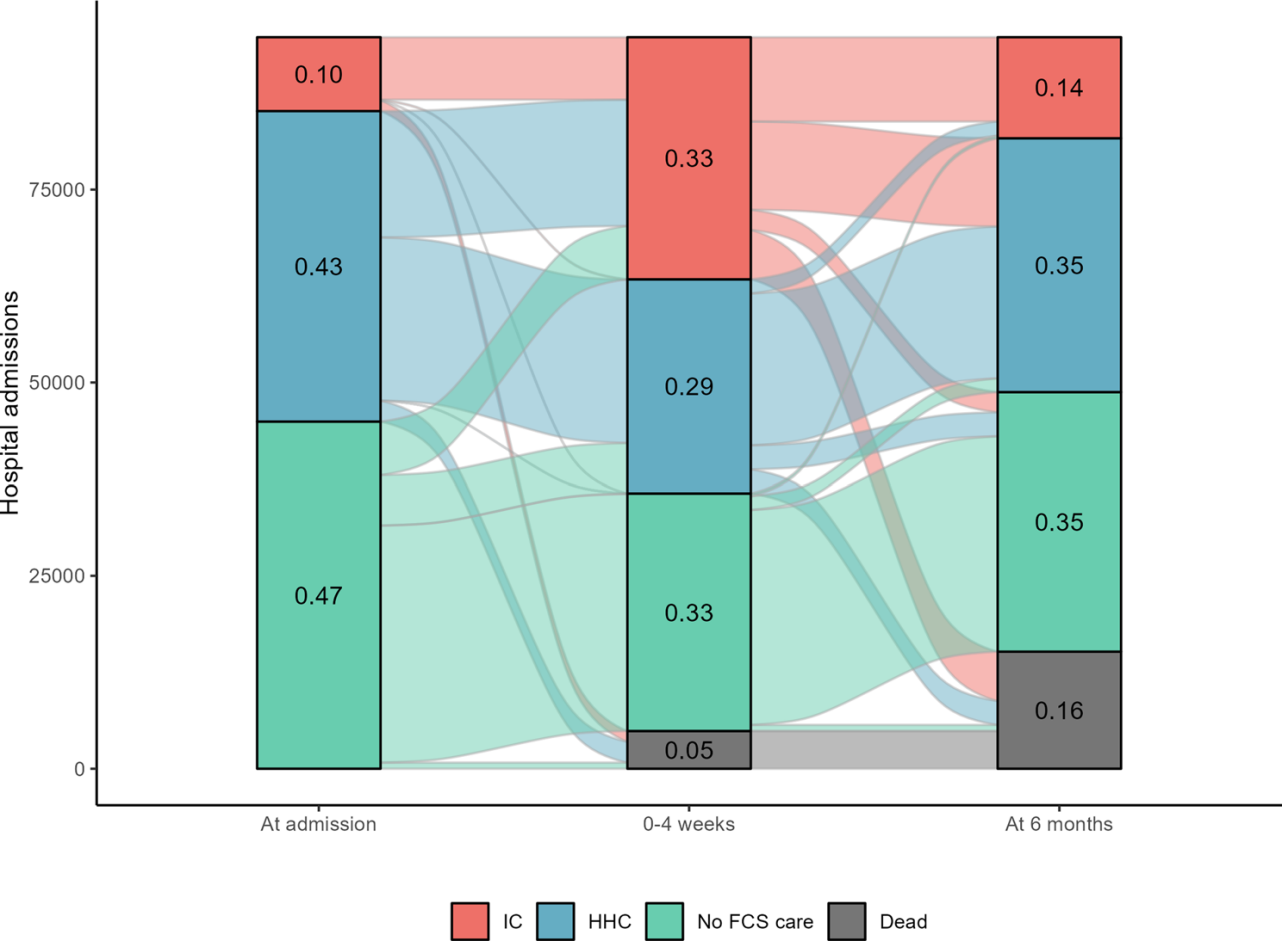
Number of hospital admissions leading to FCS/death, at admission, 4 weeks, and 6 months post-discharge

As can be seen from Table 1 (and Supplement Table S2), acute hospitalizations occur in many segments of the older population, irrespective of patient characteristics and/or prior FCS use. Admissions from home without HHC comprised less than half (47%) of all hospital discharges, whereas 43% were admitted from home with HHC and 10% from IC (see also Fig. 2). Among admissions from home without HHC, about 35% had comorbidities, whereas 1 in 3 among those admitted from home with HHC had a poor functional status. Overall, the hospital discharges included more female patients (53%), aged 75 to 85 years (58%), with low education (82%), not living alone (53%) and from higher income households (84%).

**Table 1:** Background descriptive statistics by health-related care use at hospital admission (Total, no FCS, HHC and IC) ^a^

	Total	No FCS	HHC	IC
	*N*	*N* (%)	*N* (%)	*N* (%)
Number of discharges	94,748	44,932 (47.4)	40,239 (42.5)	9577 (10.1)
Number of patients^b^	68,803	37,151 (50.8)	28,296 (38.7.1)	7720 (10.6)
**Panel A (% of discharges)**	**%**	%	%	%
**Sex**				
*Female*	53.3	48.3	58	57.4
*Male*	46.7	51.7	42	42.6
**Age**				
*75–79*	31.7	44	21.2	18.3
*80–84*	26.8	29.8	24.1	23.7
*85–89*	22.9	18.1	27	27.8
*90 +*	18.7	8.2	27.6	30.2
**Comorbidity**				
*Comorbid*	16.5	34.7		
*No comorbidity*	31.0	65.3		
***Functional status***				
*Poor*	14.1		33.3	
*Impaired*	28.3		66.7	
Prior admission within 6 months	19.7	15.1	24.3	21.7
Readmission within 30 days	42.8	44.2	43.6	33.3
Dead or emigrated within 4 weeks	5.1	1.7	6.7	14.5
Dead or emigrated within 6 months	17.6	7.7	24.1	36.3
**Education**				
*High education*	18.3	21.8	15.1	14.9
*Low education*	81.7	78.2	84.9	85.1
**Income**				
*High household*	83.7	89.5	78.4	78.5
*Low household*	16.3	10.5	21.6	21.5
**Living situation**				
*Living alone*	47.4	35.8	58.2	56.7
*Living not alone*	52.6	64.2	41.8	43.3
**Panel B (mean)**				
*LOS*,* mean*	3.90	3.67	4.27	3.33

^a^Panel A portrays proportions, whereas Panel B portrays means. LOS = length of hospital stay. ^b^The total number of patients is lower than the sum of the three groups, as patients may appear in more than one group. Consequently, the row percentages sum to > 100%

Supplement Table S2 shows that the largest diagnostic group was heart disease (25,946 discharges out of 94,748 discharges (27%)), followed by infections (24,374 discharges, 26%), whereas the smallest group was cerebrovascular disease (5,726 discharges, 6%). The patients diagnosed with infections exhibited the lowest functional status and had most comorbidity, whereas the highest functional status and least comorbidity was observed for patients with heart disease. The 30 days readmission rate was 43%, most profound for heart disease (46%), and lowest among geriatric patients (35%). Table S2 shows that the control group is largely like the other diagnostic groups with respect to patient characteristics, and the shares are neither consistently lower nor higher in comparison.

Figure 2 (and Table 1) shows that 53% of the sample were already recipients of FCS (either HHC or IC) at the time of hospitalization. Figure 2 further shows that within 4 weeks post-discharge, the shares decreased to 29% for HHC and increased to 33% for IC. At 6 months, the IC uptake was reduced to 14% but the HHC uptake had increased to 35%. Altogether, 5% died within the first 4 weeks, whereas 16% had died at 6 months. Emigration was negligible.

For those with *no prior FCS* (non-FCS users, *N* = 44,139) who survived the first 4 weeks, 6,509 (14%) transitioned to HHC and 6,910 (15%) to IC within 4 weeks. At 6 months, 41,487 had survived, and among these, 1,478 (4%) received HHC and 7,594 (18%) IC. For *prior HHC users* (*N* = 37,536) who survived the 4 weeks, we observed 16,366 (44%) transitions to IC within 4 weeks. At 6 months, 30,528 of these had survived and 6,471 (21%) had transitioned to IC.

In summary, the initial descriptive statistics suggest relatively high FCS transition rates among older individuals experiencing acute hospitalizations. The initial increase is especially evident for IC. For HHC, the pattern is less clear, likely due to a substitution between HHC and IC in the longer-term. There is also variation related to hospitalization and patient characteristics.

### Modelled results

#### Transition into FCS

**Fig. 3:**
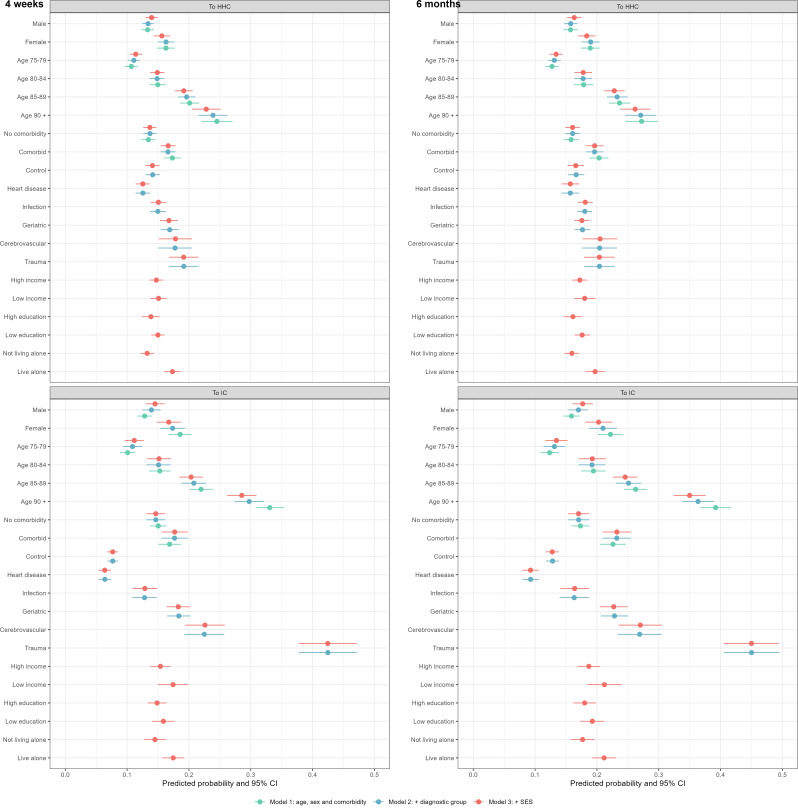
Predicted probabilities (CI) of HHC or IC transitions for non-FCS-users, 4 weeks and 6 months. Note: Green dots: Model 1, adjusted for age, sex and comorbidity. Blue dots: Model 2, Model 1 plus ICD-10 diagnosis groups, previous admission with the same diagnostic group within 6 months and lengths of hospital stay (LOS). Red dots: Model 3, Model 2 plus income, education and living situation. Estimates for previous admission with the same diagnostic group within 6 months and LOS are not shown. CI = 95% confidence intervals

Transitions into FCS are assessed for those with no FCS use on hospital admission (*N* = 44,932). Figure 3 presents the results from three different models, for two different outcomes (HHC and IC, reference = no FCS) and two time periods (at 4 weeks and 6 months after discharge). As stated previously, the models build on one another, going from basic (Model 1, green), to include also characteristics related to the hospital stay (Model 2, blue), to the full and final model (Model 3, red). In the following, we only report on estimates from the full model. Estimates (in odds ratio) from all three models are shown in Supplement Table S3.1 and S3.2.

Females (predicted probability 0.17, 95% confidence interval (CI) = 0.15–0.19) had a marginally higher probability of *transitioning into IC* within 4 weeks than males (0.15, CI = 0.13–0.16). Furthermore, patients with comorbidity had a higher probability (0.18, CI = 0.16–0.20) than those with no comorbidity (0.15, CI = 0.13–0.16). The highest probability of transitioning into IC was found for patient diagnosed with trauma (0.42, CI = 0.38–0.47). Conversely, heart disease was associated with the lowest transition probability (0.06, CI = 0.05–0.07), and this was also lower than what was observed for the control group (0.08, CI = 0.07–0.09). Additionally, a high probability of transitioning into IC was found for patients living alone (0.17, CI = 0.16–0.19). Smaller differences were also observed for patients’ education level and income.

Similar patterns were observed also for *transitions into HHC*, with increased risks observed for females (0.16, CI = 0.14–0.17), patients with comorbidity (0.17, CI = 0.15–0.18) and living alone (0.17, CI = 0.16–0.19) and patients diagnosed with trauma (0.19, CI = 0.17–0.22). For outcomes at 6 months, we saw similar results, except that the probabilities were marginally larger. An exception was that patients with cerebrovascular disease had the same probability of transitioning into HHC as had trauma patients (see Fig. 3 and Supplement Table S3.1 and S3.2). The robustness check including hospital fixed effects did not change the results in a notable manner for transitions into either HHC or IC within 4 weeks or at 6 months after hospital discharge (Figure S4).

#### Transition within FCS

Transitions within FCS apply to those who are HHC-users on hospital admission (*N* = 40,239), and thus at risk for an IC transition. Similar models as those previously described are applied, but as we have a functional status measure for this group, we model functional status instead of comorbidity for this sample.

**Fig. 4:**
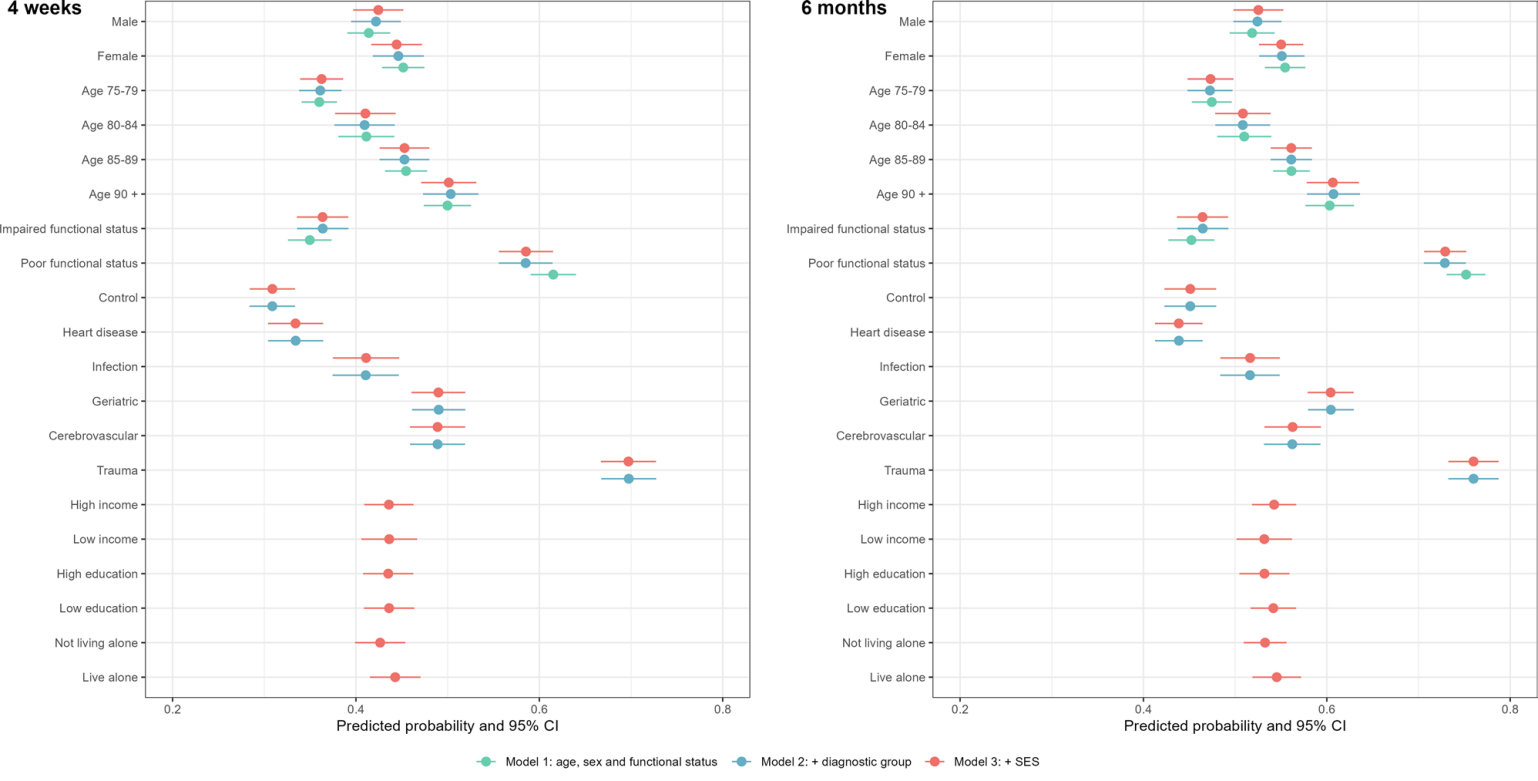
Predicted probabilities (CI) of IC transitions for HHC-users, 4 weeks and 6 months. Note Green dots: Model 1, adjusted for age, sex and functional status. Blue dots: Model 2, Model 1 plus ICD-10 diagnosis groups, previous admission with the same diagnostic group within 6 months, and LOS. Red dots: Model 3, Model 2 plus income, education and living situation. Estimates for previous admission with the same diagnostic group within 6 months and LOS are not shown. CI = 95% confidence intervals

Figure 4 (and Supplement Table S4.1 and S4.2) shows that the patterns observed were largely similar as to those we observed for new transitions (cf. above). The probability of an IC transition was, however, higher for HHC-users than for those with no prior FCS use. For transitions into IC within 4 weeks, the marginal predictions were 0.44 (CI = 0.42–0.47) for females, 0.59 (CI = 0.56–0.61) for patients with poor functional status, and 0.44 (CI = 0.42–0.47) for patients living alone. Patients diagnosed with trauma had the highest risk (0.70, CI = 0.67–0.73) whereas patients with heart disease had the lowest risk (0.33, CI = 0.30–0.36). At six months, we observed very similar estimates and patterns (see Fig. 4). Compared to the other characteristics, poor functional status and being diagnosed with trauma were more strongly associated with IC transitions for HHC-users than for those with no prior FCS use. Additionally, no substantial differences were observed among HHC-users for IC transition risks by patients’ education or income. Our first robustness check including hospital fixed effects gave very similar results (Figure S5). Our second robustness check, where we excluded prior hospitalizations (*N* = 22,990), also gave virtually identical results (Figure S6).

### Moderating factors

**Fig. 5:**
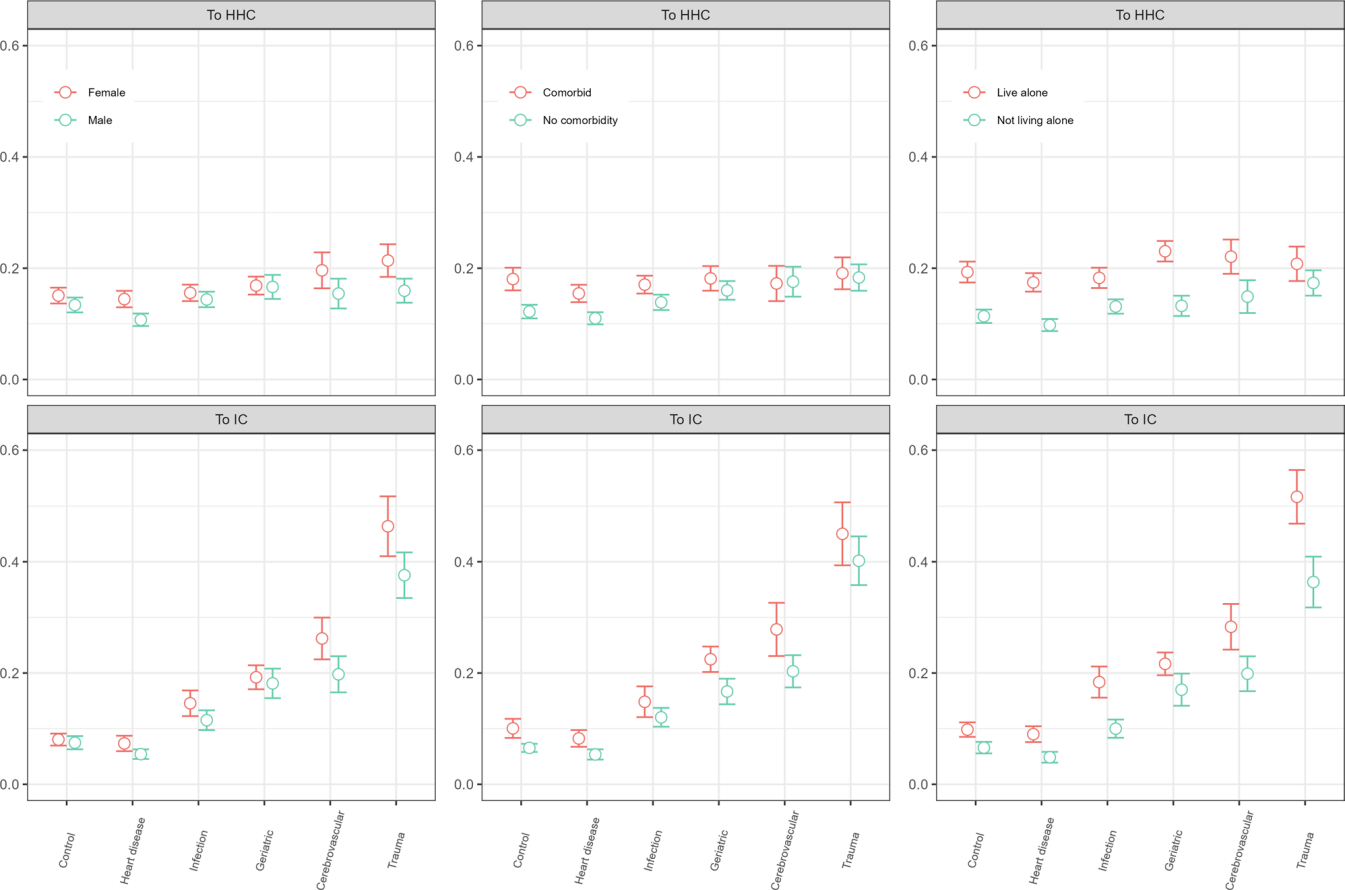
Predicted probabilities (CI) of HHC or IC transitions by diagnoses and patient characteristics, 4 weeks. Note Estimates are based on the full model (Model 3) plus interaction term between the diagnostic group and the selected characteristics (sex, comorbidity and living situation). CI = 95% confidence intervals

Figure 5 (and Supplement Table S5) shows the estimates of the diagnostic groups on transitions to HHC or IC modified by sex, comorbidity and living alone. Whereas the probability for transitioning into IC for trauma patients was 0.42 (CI = 0.38–0.47) overall, the probability was 0.46 (CI = 0.41–0.52) for female trauma patients when we allowed the associations to vary between men and women. We also saw variations according to comorbidity status by diagnostic group, and here the results differed across HHC and IC. Greatest variation across diagnostic groups was observed for patients’ living situation, with patients living alone consistently having higher uptake of both HHC and IC. Supplementary Figure S1 shows that the pattern was fairly similar also at 6 months, and thus consistent across the two time periods examined. Statistically non-significant results were largely observed for the patient characteristics education and income, across HHC and IC, for both time periods (not shown).

For HHC-users at hospital admission, the probability of transitioning into IC was much higher for those with poor functional status across all acute events, both within 4 weeks and at 6 months after hospital discharge (Supplement Table S6 and Supplement Figures S2 and S3). The association was, however, weaker (or not present) for women versus men and living alone versus not living alone.

## Discussion and concluding remarks

Irrespective of patient characteristics and/or prior FCS use, acute hospitalizations occur in many segments of the older population and are associated with an increase in the *subsequent* uptake of FCS. In the short-term, the uptake of IC goes up, whereas the uptake of HCC declines. In the longer-term, IC is reduced and substituted largely by HHC, although the IC uptake remains at a higher level than what was observed prior to the hospitalization. The associated IC use is fairly pronounced, given that a marked share dies within the first 6 months and thus do not need further IC.

FCS uptake post-hospitalization appears to depend on whether the older individual resided at home with or without HHC prior to the hospital admission, and at what point in time the uptake was assessed (**RQ**_**1**_). Furthermore, the acute event is associated with an increased need for IC for those already in receipt of HHC prior to the acute event (**RQ**_**2**_). Lastly, marked heterogeneity in FCS uptake post-hospitalization is observed by diagnosis, comorbidity and/or functional status, and characteristics related to the hospital admission itself, whereas sociodemographic patient characteristics appear to matter less, except for living alone (**RQ**_**3**_).

To our knowledge, scarce research includes both home health and institutionalized care in examinations of patterns of FCS utilisation after acute hospitalizations of older persons. The studies we have been able to locate, stems largely from contexts where private arrangements are more common than what we see in the Norwegian context, such as the US, thus making comparisons challenging. Nevertheless, our results pertaining to **RQ1** align well with research that finds an increased uptake of IC after acute hospitalizations in older individuals [12], especially for trauma, cerebrovascular disease and geriatric conditions. In line with earlier work [42], trauma was found to be the condition most strongly affecting subsequent FCS uptake, and the impact was particularly pronounced for IC. Injuries resulting from falls comprise a major share in this group, and since falls can be prevented [43], more resources directed at primary (e.g., preventing osteoporosis) or secondary and tertiary prevention measures (e.g., avoiding unnecessary polypharmacy that may impact balance) at home, in IC, as well as in hospitals appears warranted. Use of allied health professions such as physical or occupational therapists in the home setting may be able to improve individual physical functioning and/or enhance functional status by ensuring a safer home environment.

The increased uptake of IC associated with cerebrovascular disease is not surprising, as these diseases typically have a profound impact on functional level (cognitive and physical) and thus the need for FCS. Prevention is, however, also possible for this disease group. Similarly, also the marked uptake of IC for persons admitted for geriatric conditions aligns well with prior research which found that also hip fractures, reduced mobility, and multiple comorbidities were significant predictors for IC [44]. Whereas we find that both cognitive and physical functioning matter for subsequent FCS uptake, other studies find the physical aspect to be most important for IC admission [18]. Nevertheless, as cognitive problems represent important risk factors for functional decline during hospitalization in older patients, an assessment of cognitive status of all hospitalized older adults both on admission and at regular intervals during hospitalization appears warranted.

Surprisingly, we observed that the uptake of FCS was equally low (or lower) among those admitted with heart conditions as compared to our control group. According to national guidelines, acute heart disease and symptoms such as chest pain frequently require specialized care, and nearly all persons with such symptoms are thus admitted to hospitals, regardless of functional level. This is different than for infections. Fit patients with infections can be treated with antibiotics at home or in IC, so only sepsis or serious infections typically lead to an acute hospital admission. In patients living with frailty, however, even a mild infection such as that of the lower urinary tract may lead to delirium and thus hospitalization due to acute functional decline. Due to this selection, it is thus not surprising that FCS uptake was high among patients admitted for infectious diseases.

For **RQ2**, we have identified few studies that examine discharges to IC for patients already in receipt of HHC at hospital admission. Our results seem, however, plausible, and underscore that acute events appear to change care needs also among individuals in receipt of HHC, who at least in Norway are quite frail [23], with marked cognitive and/or functional limitations. At the same time, although our findings suggest that functionally frail older patients may require IC for more intensive rehabilitation or other health services as a transient placement to enable a subsequent recovery at home, the share relying on IC was higher at 4 weeks than at 6 months. Future efforts directed at improving post-acute care, particularly in high-risk populations, should not only consider the immediate hospital discharge, but also transitions within the care system, such as between IC and HHC as well as between HHC and IC. A review documents that shorter IC stays tend to be associated with reduced physical functioning [16], whereas this is observed to a lesser extent for declines in cognitive functioning [45]. This is to be expected as it may be easier to enhance physical (through, for instance, rehabilitation) than cognitive functioning after an acute event. Furthermore, previous studies have shown that only a minority of patients who transfer to (long-term) IC return to pre-hospital daily functional status [6, 20]. While most of our results align with this finding, our results for prior HHC-users nevertheless indicate that around half *do* return home between 4 weeks and 6 months. This is encouraging, given that most older adults prefer to age in place when possible [19]. It should nevertheless be noted that older individuals admitted acutely to hospitals comprise a frail group, and a notable share dies within 6 months. In our sample, this pertained to 5% at 4 weeks and 16% at 6 months, in line with estimates reported elsewhere [46]. This means that the population is more select at 6 months than at 4 weeks, as illustrated in Fig. 2. Readmissions to hospitals may also be an indicator of frailty, and 4 in 10 in our sample were readmitted to hospitals within 4 weeks, which is higher than what is commonly reported [10]. It should be noted, however, that most other estimates only apply to re-hospitalizations from IC, and do not include rehospitalizations from home, with or without HHC, as we do here. However, compared to non-institutionalized older persons, IC residents tend to have poorer health, including higher rates of dementia, stroke and severe mental illness [45] which in turn results in an increased use of health services, such as hospital and emergency department admission, primary care contact and use of out of hours services [10].

Research related to **RQ**_**3**_ that pertains to *HHC transitions* are also somewhat scarce, but our findings align well with results showing that older adults discharged to HHC continue to require care for 3 months or more [30]. More research exists on *IC transitions*. Here our results are largely in line with existing research, in that there is variation across characteristics related to the hospitalization itself as well as across patient characteristics [7, 45]. An earlier review shows, for instance, that IC uptake is largely based on underlying cognitive and/or functional impairment, and associated lack of support and assistance in daily living [7]. Irrespective of frailty status, older people with more social resources may be able to forestall IC placements longer than individuals with fewer resources [6, 7]. This aligns well with our findings on higher uptake among persons living alone, and especially women, which is found also elsewhere [45]. A recent systematic review and meta-analysis of predictors of IC placement following acute hospitalizations found, however, that social support was rarely considered [12].

More resources provided to FCS are likely to contribute to better quality care while at the same time reducing the overall societal costs of treatment of older patients [11, 13, 47], as higher social care expenditure and greater availability of IC have been found to be associated with fewer hospital readmissions and delayed discharges [11], reduced hospital stays, and reduced expenditure on care services [13, 47]. To assess the role of FCS availability and variability in more detail, comparative cross-national studies are needed. However, within existing resource frameworks, a better coordination, integration and collaboration between hospitals and the FCS as well as between IC and HHC within the FCS, could likely improve care trajectories.

### Limitations

While this study has several strengths, such as the use of high-quality register data encompassing the entire Norwegian population of older individuals with select diagnoses, functional measures and/or comorbidities prior to the acute hospital event, as well as sociodemographic characteristics, we also document robust findings irrespective of model specification. Some limitations should, however, be noted. First, the descriptive nature implies that we can merely report on associations between acute hospitalizations and subsequent care use, which prevents us from making any causal claims. Thus, we are unable to resolve inherent issues related to selection, with possible consequences for the validity and generalizability of our findings, as this warrants different data and different designs. Second, we lack functional measures and measures of cognitive decline for individuals who are admitted from home without HHC. We also lack information on the availability of social support, although individuals’ living situation is accurately recorded. Third, the geriatric disease group we apply is quite broad, and we did not have sufficient power to examine the included diagnoses individually. Many studies that examine diseases in more detail find that the specific disease matters. A review shows, for instance, that delirium is an important predictor for the risk of long-term IC after hospitalization, independent of important confounders, such as age, sex, comorbidity and dementia [48]. Future studies could attempt to disentangle the specific diagnostic groups to a larger extent than what was possible here. Fourth, in line with most studies in this area [22], we are not able to distinguish between different types of IC, such as for instance skilled nursing facilities and traditional, long-term nursing home care. All types of IC were thus grouped together, although we acknowledge that the exact type of facility may have a bearing on results [37]. Future studies should thus attempt to make such a distinction. Also, the types and amounts of awarded HHC may matter, and future studies should attempt to include such information. Last, a discharge with worse function does not preclude future improvements [30], but our outcomes pertain to a *rise* in care needs. Thus, the few individuals who appeared to functionally improve (as proxied by subsequent FCS uptake) after hospitalization were grouped in the no change group in line with the functional recovery definition stating that proxying functional recovery by need for services may allow for a pragmatic and clinically meaningful definition [30]. As is shown in Fig. 2, this pertains to few individuals. However, future studies could examine this group in more detail, to possibly learn from successful hospital discharges, both in the short- and long-term.

### Concluding remarks

We have provided data on a full-population sample of older adults admitted acutely to hospital with select diagnoses and examined transfers to either HHC or IC in the short- and longer-term, thus adding to the scarce evidence base that exists. We found that acute hospital admissions are frequent among older individuals, irrespective of prior FCS uptake. In fact, more than half of admitted patients were already recipients of FCS at hospital admission. As existing studies often exclude this group, results may be biased. Acute admissions are, however, strongly associated with *subsequent* FCS uptake, but there is marked heterogeneity by diagnosis, comorbidity and/or functional status and patients’ living situation.

Population ageing and strained public resources will challenge the future provision of sustainable, safe, complex, and high-quality health and care services. More knowledge of the various pathways into and out of hospitals is vital for current and future FCS planning and policy development to enable older people to remain as independent as possible [4]. Research on the temporality and modes (HHC vs. IC) of FCS uptake is warranted to discern if the increased utilization after acute episodes is temporary or long-lasting, and if policy measures, such as interventions to prevent hospital admissions or more intense rehabilitation in the aftermath, could reduce long-term FCS needs, either at home or in institutions. Importantly, studies with causal designs are scarce, and should be encouraged. Lastly, future research should include hospital setting frailty measures to improve predictions of future FCS needs following acute episodes, and studies are warranted to examine if more can be done within IC to improve the medical knowledge to prevent as many hospital (re)admissions older individuals from IC.

## Supplementary Information

Below is the link to the electronic supplementary material.

Supplementary Material 1

## Data Availability

The data that support the findings of this study are available from the respective registers, but restrictions apply to the availability of these data. Consequently, they are not publicly available. They are available from Helsedata (see [https://helsedata.no/en/] upon reasonable request and with adequate permissions. The first author will assist in this process.

## Footnotes

1 We define FCS care services that entail an element of health care, provided within the municipal care setting either at home (HHC) or in short-, medium-, or long-term institutions (IC). Vast variations exist in the terms used to denote IC, such as ‘care homes’, ‘care facilities’, skilled nursing facilities’, ‘nursing homes’, ‘institutional long-term care’, and ‘post-acute care facilities’. We are unable to distinguish between these different arrangements in our data and use the term IC for all or any of these variants throughout the paper.

2 Hospital (re)admissions may be a substitute/competing risk for FCS for certain conditions, but also result in (increased) FCS uptake.

3 Hospital readmissions fall outside the scope of this article, and as the literature is quite extensive, only a few examples are provided.

4 We wanted to avoid calculating these events twice. As it only affected around 2% of all observations, it is highly unlikely that a shorter time frame would change the overall results in a significant manner.

5 According to the Norwegian Directorate of Health, the median life expectancy for long-term residents aged 50 or above in IC in Norway is as low as 1.3–1.6 years (www.helsedirektoratet.no/rapporter/kommunale-helse-og-omsorgstjenester-i-2023/utvalgte-temaer/gjennomsnittlig-botid-pa-langtidsopphold-i-institusjon).

6 Planned admissions and out-patient consultations were not included.

7 Unfortunately, the description of the coding of functional status is only available in Norwegian, see www.helsedirektoratet.no/veiledere/registrering-av-iplos-data-i-kommunen/om-funksjonsvurdering-og-skarinndeling/beregning-av-delmal-og-samlemal.

8 Those with missing education were assigned to the *low education category*.

9 In short, the EU-60-scale defines low income as households with an income below 60% of the median after-tax income per consumption unit in the household. The latter is calculated using the EU-scale, where the first adult is given the value 1, additional adults are given the value 0.5, and each child is given the value 0.3.
