# Supplementary material for: Institutionalization and home health care after acute hospitalizations of older persons in Norway
Source: BMC Health Serv Res. 2025 Dec 17;26:100. doi: 10.1186/s12913-025-13851-x (PMC12822220; doi:10.1186/s12913-025-13851-x)
Supplement: Supplementary file 1 — Supplementary Material 1 [file 12913_2025_13851_MOESM1_ESM.docx]

# Supplementary materials

## Figures

**Figure S1. Predicted probabilities (CI) of *HHC or IC* *transitions* by diagnoses and patient characteristics, *6 months***

**Figure S2. Predicted probabilities (CI) of *IC* *transitions* for HHC-users by diagnoses and patient characteristics, *4 weeks***

**Figure S3. Predicted probabilities (CI) of *IC* *transitions* for HHC-users by diagnoses and patient characteristics, *6 months***

**Figure S4.** **Predicted probabilities (CI) of *HHC or IC* *transitions* for non-FCS-users, 4 weeks and 6 months (*with hospital fixed effects*)**

**Figure S5. Predicted probabilities (CI) of *IC* *transitions* for HHC-users, 4 weeks and 6 months (*with hospital fixed effects*)**

**Figure S6. Predicted probabilities (CI) of *IC* *transitions* for HHC-users*,* 4 weeks and 6 months (*excluding prior hospitalizations*)**

## Tables

Table S1: List of select diagnostic groups with ICD-10 codes

Table S2: Background descriptive statistics by diagnostic group for the initial sample at hospital admission

Table S3.1: Odds ratios (OR), standard errors (SE), and p-values of *transitions into FCS (HHC or IC) within 4 weeks*, three different models

Table S3.2: Odds ratios (OR), standard errors (SE), and p-values of *transitions into FCS (HHC or IC) at 6 months*, three different models

Table S4.1: Odds ratios (OR), standard errors (SE), and p-values of *transitions into IC within 4 weeks*, three different models

Table S4.2: Odds ratios (OR), standard errors (SE), and p-values of *transitions into IC at 6 months*, three different models

Table S5: Odds ratios (OR), standard errors (SE), and p-values *of transitions into FCS (HHC or IC)* *within 4 weeks and at 6 months* for a full model with interactions

Table S6: Odds ratios (OR), standard errors (SE), and p-values of *transitions into IC within 4 weeks and at 6 months* for a full model with interactions


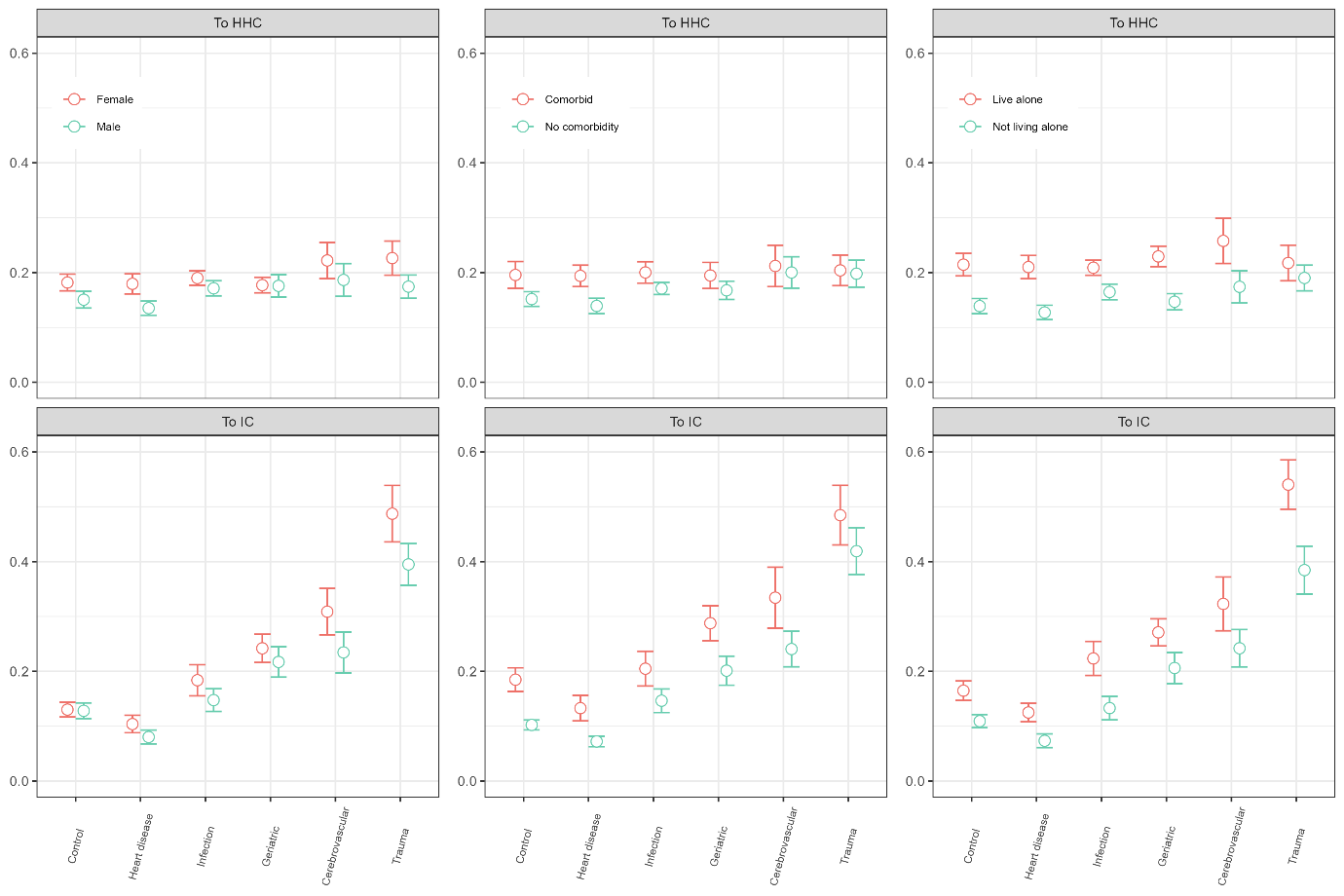


**Figure S1: Predicted probabilities (CI) of *HHC or IC transitions* by diagnoses and patient characteristics, *6 months.*** Note: Estimates are based on the full model (Model 3) plus interaction terms between the diagnostic group and patient characteristics (sex, comorbidity and living situation). CI = 95% confidence intervals.


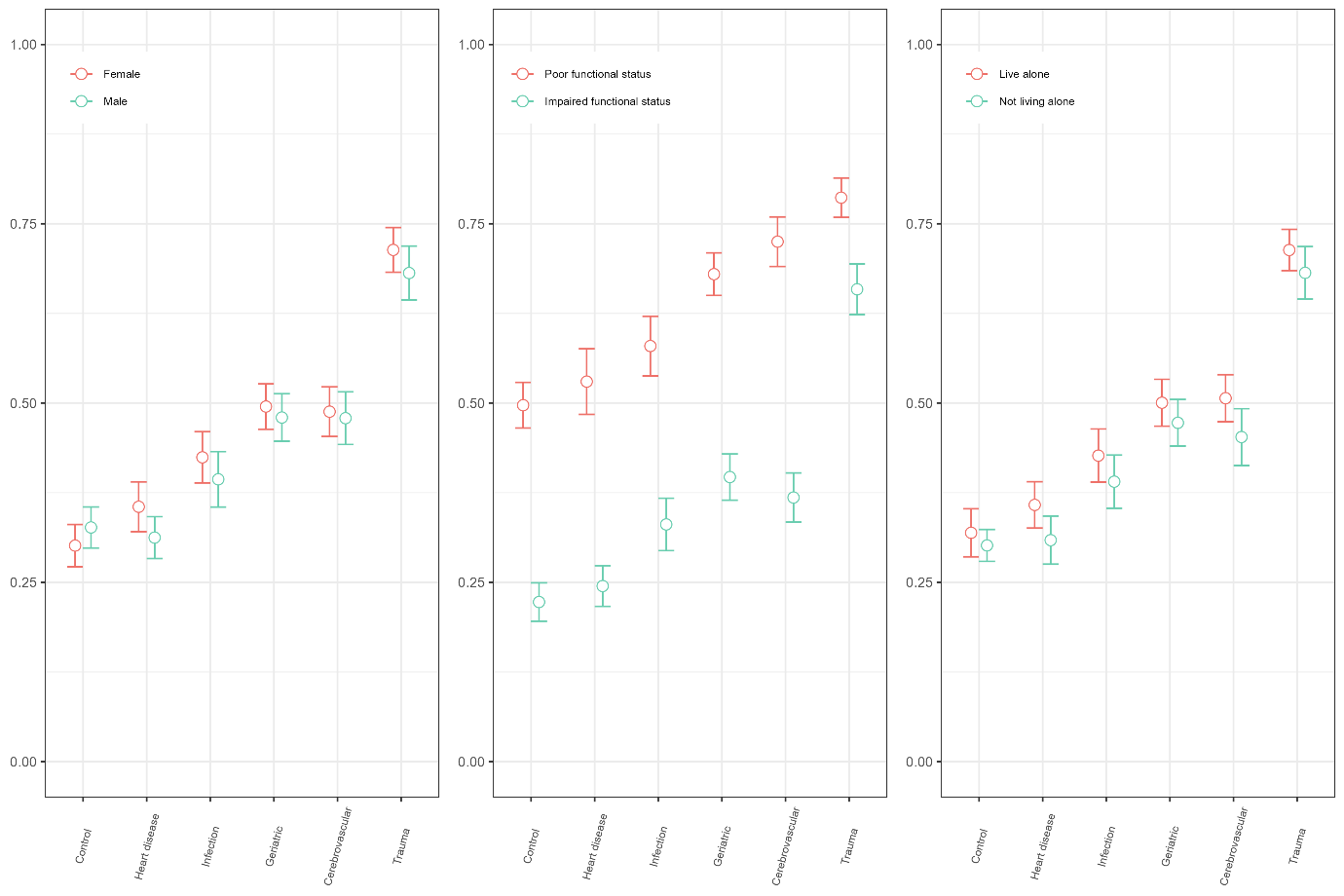


**Figure S2: Predicted probabilities (CI) of *IC transitions* for HHC-users by diagnoses and patient characteristics, *4 weeks*.** Note: Estimates are based on the full model (Model 3) plus interaction terms between the diagnostic group and patient characteristics (sex, functional status and living situation). CI = 95% confidence intervals.

**
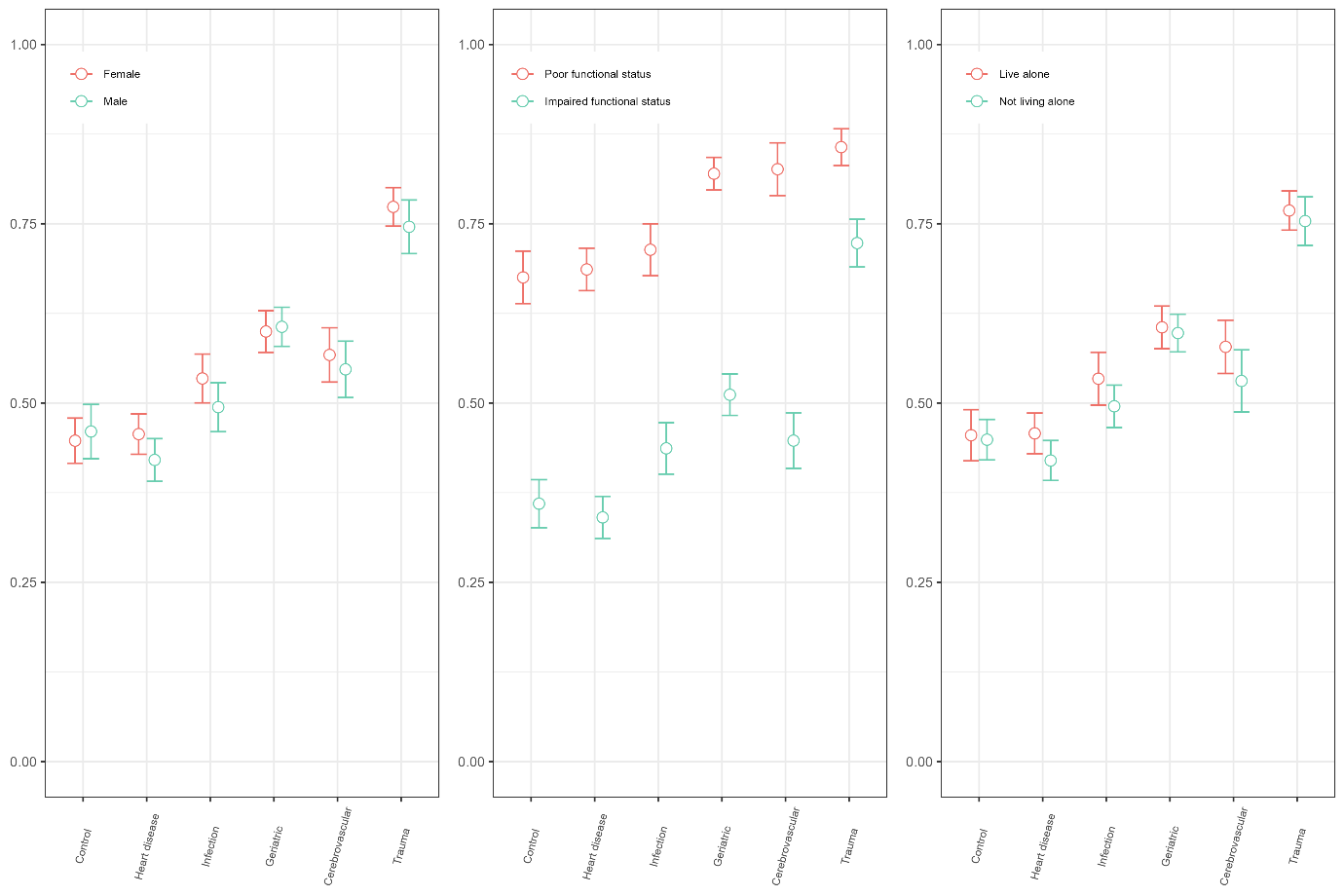
Figure S3: Predicted probabilities (CI) of *IC* *transitions* for HHC-users by diagnoses and patient characteristics, *6 months*.** Note: Estimates are based on the full model (Model 3) plus interaction term between the diagnostic group and patient characteristics (sex, functional status and living situation). CI = 95% confidence intervals.


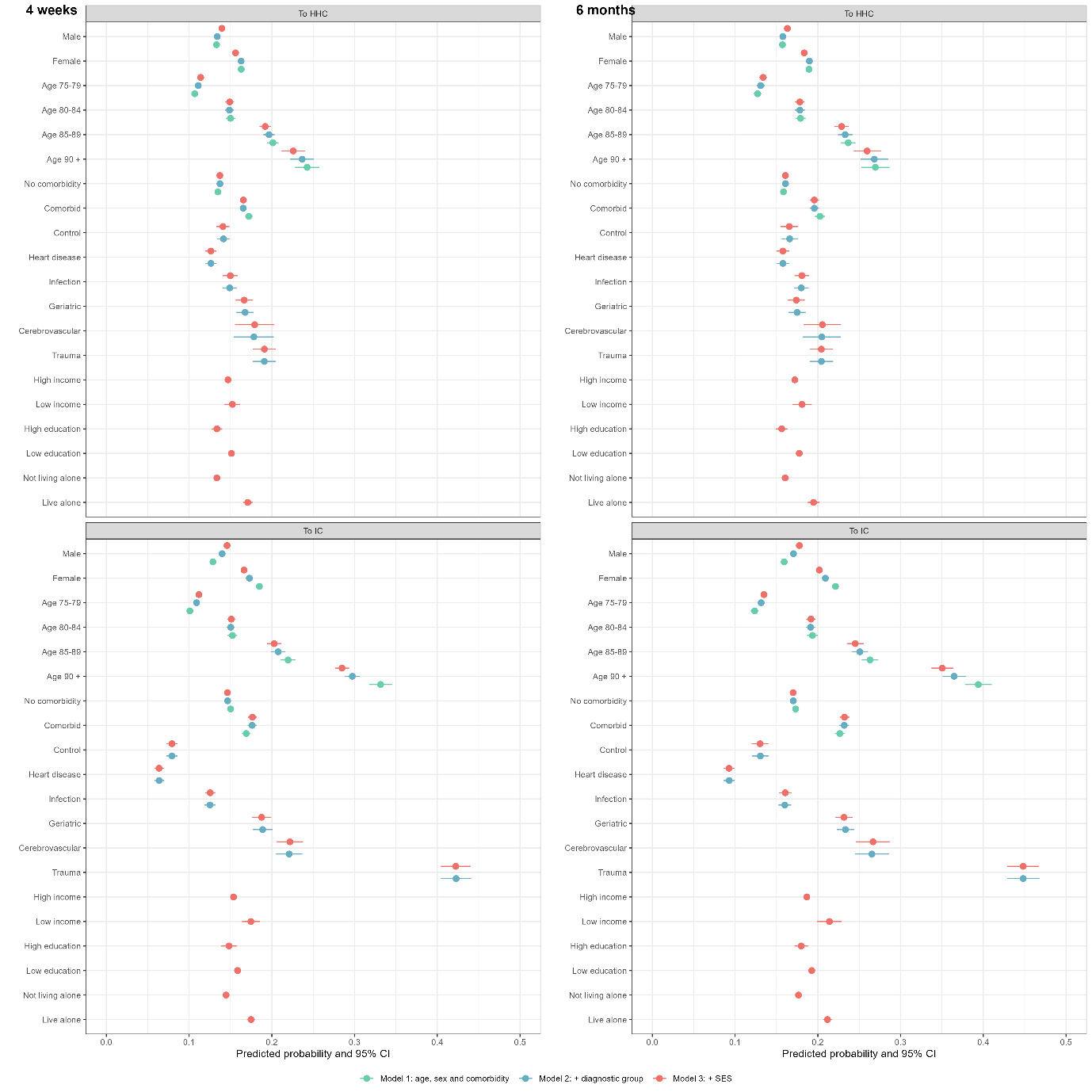


**Figure S4: Predicted probabilities (CI) of *HHC or IC transitions* for non-FCS-users, 4 weeks and 6 months (*with hospital fixed effects*).** Note: Green dots: Model 1, adjusted for age, sex and comorbidity. Blue dots: Model 2, Model 1 plus ICD-10 diagnostic group, previous admission with the same diagnostic group within 6 months and lengths of hospital stay (LOS). Red dots: Model 3, Model 2 plus income, education and living situation. Estimates for previous admission with the same diagnostic group within 6 months and LOS are not shown. CI = 95% confidence intervals.


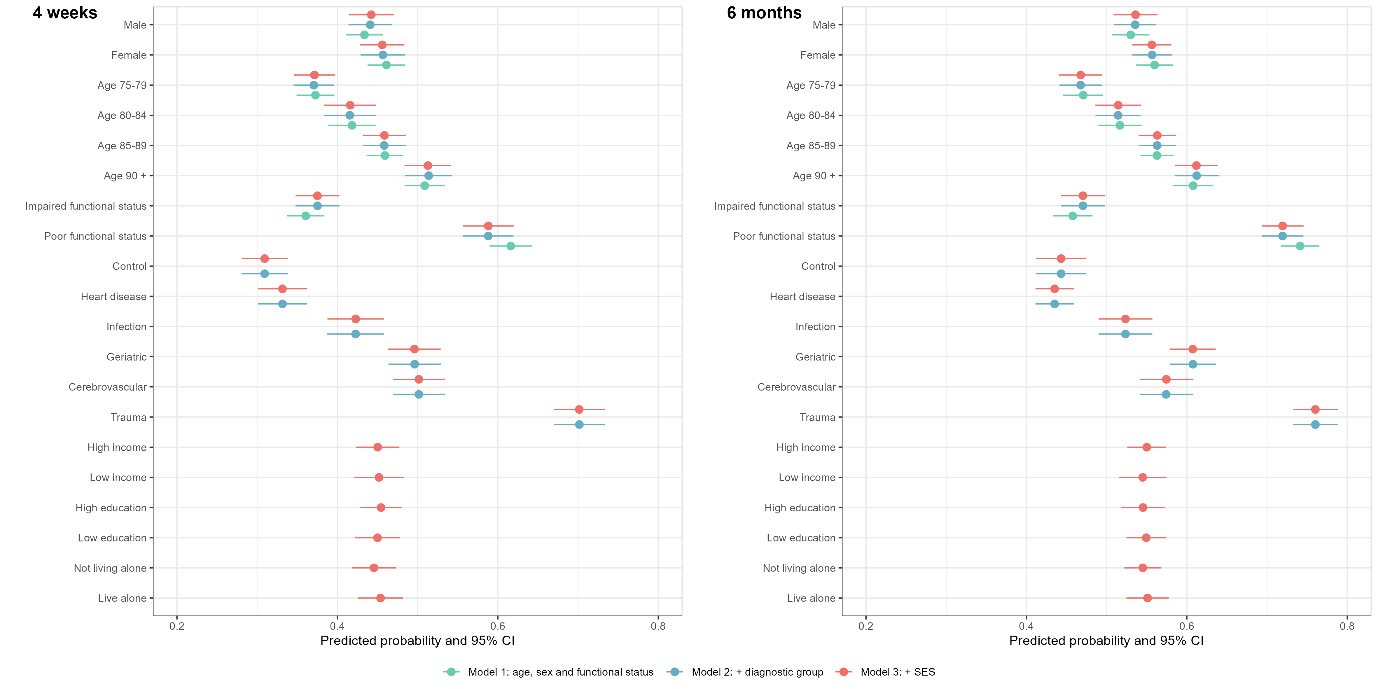


**Figure S5: Predicted probabilities (CI) of *IC* *transitions* for HHC-users, 4 weeks and 6 months (*with hospital fixed effects*).**

Note: Green dots: Model 1, adjusted for age, sex and comorbidity. Blue dots: Model 2, Model 1 plus ICD-10 diagnostic group, previous admission with the same diagnostic group within 6 months and lengths of hospital stay (LOS). Red dots: Model 3, Model 2 plus income, education and living situation. Estimates for previous admission with the same diagnostic group within 6 months and LOS are not shown. CI = 95% confidence intervals.


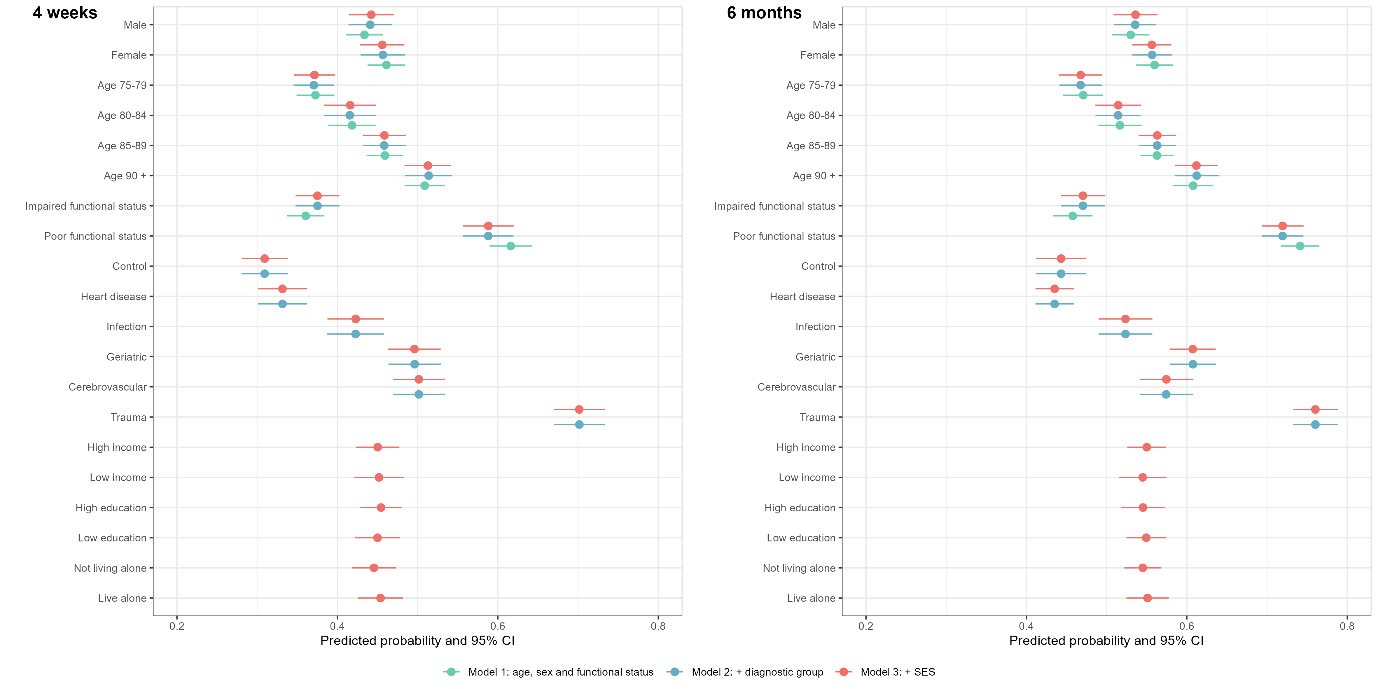


**Figure S6: Predicted probabilities (CI) of *IC* *transitions* for HHC*-*users, 4 weeks and 6 months (*excluding prior hospitalizations*).** Note: Green dots: Model 1, adjusted for age, sex and comorbidity. Blue dots: Model 2, Model 1 plus ICD-10 diagnostic group, previous admission with the same diagnostic group within 6 months and lengths of hospital stay (LOS). Red dots: Model 3, Model 2 plus income, education and living situation. Estimates for previous admission with the same diagnostic group within 6 months and LOS are not shown. CI = 95% confidence intervals.

Table S1: List of select diagnostic groups with ICD-10 codes.

| **Diagnostic group** | **ICD-codes** | **Diagnosis** |
| --- | --- | --- |
| **Infection** | A41  A46  A49  J10  J12  J13  J14  J15  J18  J22  J44.0  K80  L02  N10  N30  N39 | Other sepsis  Erysipelas  Bacterial infection of unspecified site  Influenza due to identified seasonal influenza virus  Viral pneumonia. not elsewhere classified  Pneumonia due to Streptococcus pneumoniae  Pneumonia due to Haemophilus influenzae  Bacterial pneumonia. not elsewhere classified  Pneumonia. organism unspecified  Unspecified acute lower respiratory infection  Chronic obstructive pulmonary disease with acute lower respiratory infection  Cholelithiasis  Cutaneous abscess. furuncle and carbuncle  Acute tubulo-interstitial nephritis  Cystitis  Other disorders of urinary system |
| **Heart disease** | I20  I21  I26  I30  I31  I32  I33  I34  I35  I36  I37  I38  I39  I40  I41  I42  I43  I44  I45  I46  I47  I48  I49  I50  I51  I52  R55 | Angina pectoris  Acute myocardial infarction  Pulmonary embolism  Acute pericarditis  Other diseases of pericardium  Pericarditis in diseases classified elsewhere  Acute and subacute endocarditis  Nonrheumatic mitral valve disorders  Nonrheumatic aortic valve disorders  Nonrheumatic tricuspid valve disorders  Pulmonary valve disorders  Endocarditis. valve unspecified  Endocarditis and heart valve disorders in diseases classified elsewhere  Acute myocarditis  Myocarditis in diseases classified elsewhere  Cardiomyopathy  Cardiomyopathy in diseases classified elsewhere  Atrioventricular and left bundle-branch block  Other conduction disorders  Cardiac arrest  Paroxysmal tachycardia  Atrial fibrillation and flutter  Other cardiac arrhythmias  Heart failure  Complications and ill-defined descriptions of heart disease  Other heart disorders in diseases classified elsewhere  Syncope and collapse |
| **Cerebrovascular disease** | I60  I61  I62  I63  I64  I65  I66  I67  I68  I69 | Subarachnoid haemorrhage  Intracerebral haemorrhage  Other nontraumatic intracranial haemorrhage  Cerebral infarction  Stroke. not specified as haemorrhage or infarction  Occlusion and stenosis of precerebral arteries. not resulting in cerebral infarction  Occlusion and stenosis of cerebral arteries. not resulting in cerebral infarction  Other cerebrovascular diseases  Cerebrovascular disorders in diseases classified elsewhere  Sequelae of cerebrovascular disease |
| **Trauma** | M80  S06  S22  S32  S42  S72  S82 | Osteoporosis with pathological fracture  Intracranial injury  Fracture of rib(s). sternum and thoracic spine  Fracture of lumbar spine and pelvis  Fracture of shoulder and upper arm  Fracture of femur  Fracture of lower leg. including ankle |
| **Geriatric** | E43  E86  E87  F01  F05  F06.7  F10  G20  G30  H81  M54  R29  R32  R41.8  R42 | Unspecified severe protein-energy malnutrition  Volume depletion  Other disorders of fluid. electrolyte and acid-base balance  Vascular dementia  Delirium. not induced by alcohol and other psychoactive substances  Mild cognitive disorder  Mental and behavioural disorders due to use of alcohol  Parkinson disease  Alzheimer disease  Disorders of vestibular function  Dorsalgia  Other symptoms and signs involving the nervous and musculoskeletal systems  Unspecified urinary incontinence  Other and unspecified symptoms and signs involving cognitive functions and awareness  Dizziness and giddiness |
| **Control** | D50  E10  E11  G40  G45  I10  I11  K40  K43  K59  K92  M16  M79  N20  R07 | Iron deficiency anaemia  Type 1 diabetes mellitus  Type 2 diabetes mellitus  Epilepsy  Transient cerebral ischaemic attacks and related syndromes  Essential (primary) hypertension  Hypertensive heart disease  Inguinal hernia  Ventral hernia  Other functional intestinal disorders  Other diseases of digestive system  Coxarthrosis [arthrosis of hip]  Other soft tissue disorders. not elsewhere classified  Calculus of kidney and ureter  Pain in throat and chest |

Table S2. Background descriptive statistics by diagnostic group for the initial sample at hospital admission.^a^

|  | **Total** | **Control** | **Cerebrovascular** | **Geriatric** | **Heart** | **Infection** | **Trauma** |
| --- | --- | --- | --- | --- | --- | --- | --- |
|  | N | N | N | N | N | N | N |
| Number of discharges (N) | 94748 | 14406 | 5726 | 9795 | 25948 | 24374 | 14499 |
| Number of patients^b^ | 68803 | 12715 | 5360 | 8961 | 20450 | 19581 | 13499 |
| **Panel A (% of discharges)** | % | % | % | % | % | % | % |
| **Healthcare use at admission** |  |  |  |  |  |  |  |
| *Admitted from IC* | 10,1 | 8,2 | 7,5 | 8,9 | 5,7 | 13,1 | 16,8 |
| *Only HHC* | 42,5 | 38,4 | 32,5 | 48,9 | 36,6 | 51,2 | 41,9 |
| *No FCS use* | 47,4 | 53,4 | 60,0 | 42,2 | 57,7 | 35,7 | 41,3 |
| **Sex** |  |  |  |  |  |  |  |
| *Female* | 53,3 | 56,4 | 50,0 | 58,7 | 49,3 | 46,5 | 66,6 |
| *Male* | 46,7 | 43,6 | 50,0 | 41,3 | 50,7 | 53,5 | 33,4 |
| **Age** |  |  |  |  |  |  |  |
| *75-79* | 31,7 | 35,1 | 33,5 | 30,9 | 33,3 | 32,4 | 24,0 |
| *80-84* | 26,8 | 27,7 | 28,4 | 27,2 | 26,4 | 26,6 | 25,9 |
| *85-89* | 22,9 | 21,5 | 22,5 | 23,9 | 22,4 | 22,5 | 25,1 |
| *90 +* | 18,7 | 15,7 | 15,5 | 18 | 17,9 | 18,5 | 2,5 |
| **Functional status** |  |  |  |  |  |  |  |
| *Only HHC and poor function* | 14,1 | 10,0 | 13,6 | 19,9 | 9,0 | 18,7 | 16,0 |
| *Only HHC and impaired function* | 28,3 | 28,4 | 18,9 | 29,0 | 27,6 | 32,4 | 26,0 |
| **Comorbidity** |  |  |  |  |  |  |  |
| *No FCS use and comorbidity* | 16,5 | 16,0 | 14,1 | 11,4 | 22,7 | 16,7 | 9,7 |
| *No FCS use and no comorbidity* | 31,0 | 37,4 | 45,9 | 30,8 | 35,0 | 19,1 | 31,6 |
| Prior admission^c^ within 6 months | 19,7 | 18,5 | 9,9 | 16,1 | 28,0 | 21,1 | 9,8 |
| Readmission within 30 days | 42,8 | 42,2 | 37,8 | 35,3 | 46,7 | 44,9 | 40,2 |
| Dead or emigrated within 4 weeks | 5,1 | 2 | 6,7 | 3,7 | 4,7 | 7,5 | 5,5 |
| Dead or emigrated within 6 months | 17,6 | 11,5 | 16,4 | 14,5 | 17 | 24,1 | 16,2 |
| **Education** |  |  |  |  |  |  |  |
| *High education* | 18,3 | 18,0 | 21,0 | 20,1 | 19,1 | 16,6 | 17,6 |
| *Low education* | 81,7 | 82,0 | 79,0 | 79,9 | 80,9 | 83,4 | 82,4 |
| **Income** |  |  |  |  |  |  |  |
| *High household* | 83,7 | 84,3 | 85,0 | 83,7 | 84,8 | 84,3 | 79,7 |
| *Low household* | 16,3 | 15,7 | 15,0 | 16,3 | 15,2 | 15,7 | 20,3 |
| **Living situation** |  |  |  |  |  |  |  |
| *Living alone* | 47,4 | 46,3 | 43,7 | 51,1 | 45,2 | 45,3 | 55,0 |
| *Living not alone* | 52,6 | 53,7 | 56,3 | 48,9 | 54,8 | 54,7 | 45,0 |
| **Panel B (mean)** |  |  |  |  |  |  |  |
| *LOS, mean* | 3,90 | 2,40 | 5,10 | 3,20 | 3,90 | 4,80 | 4,00 |

^a^Panel A portrays proportions, whereas Panel B portrays means. LOS= length of hospital stay. ^b^The total number of patients is lower than the sum of the diagnostic groups, as patients may appear in more than one group. ^c^Prior admission; previous admission within 6 months within the same diagnostic group.

Table S3.1: Odds ratios (OR), standard errors (SE), and p-values of *transitions into FCS (HHC or IC) within 4 weeks*, three different models

|  | Model 1 | | Model 2 | | Model 3 | |
| --- | --- | --- | --- | --- | --- | --- |
|  | To HHC | To IC | To HHC | To IC | To HHC | To IC |
| (Intercept) | 0.10*** | 0.09*** | 0.03*** | 0.01*** | 0.02*** | 0.01*** |
|  | (0.00),[<0.01] | (0.00),[<0.01] | (0.00),[<0.01] | (0.00),[<0.01] | (0.00),[<0.01] | (0.00),[<0.01] |
| Female | 1.42*** | 1.69*** | 1.46*** | 1.60*** | 1.25*** | 1.35*** |
|  | (0.04),[<0.01] | (0.05),[<0.01] | (0.04),[<0.01] | (0.05),[<0.01] | (0.04),[<0.01] | (0.05),[<0.01] |
| Age 80-84 | 1.60*** | 1.73*** | 1.65*** | 1.87*** | 1.60*** | 1.81*** |
|  | (0.05),[<0.01] | (0.06),[<0.01] | (0.06),[<0.01] | (0.07),[<0.01] | (0.06),[<0.01] | (0.07),[<0.01] |
| Age 85-89 | 2.61*** | 3.02*** | 2.89*** | 3.77*** | 2.67*** | 3.46*** |
|  | (0.10),[<0.01] | (0.11),[<0.01] | (0.11),[<0.01] | (0.16),[<0.01] | (0.11),[<0.01] | (0.15),[<0.01] |
| Age 90 + | 4.38*** | 6.28*** | 5.19*** | 8.99*** | 4.53*** | 7.73*** |
|  | (0.21),[<0.01] | (0.29),[<0.01] | (0.27),[<0.01] | (0.49),[<0.01] | (0.24),[<0.01] | (0.43),[<0.01] |
| Comorbid | 1.43*** | 1.25*** | 1.44*** | 1.53*** | 1.46*** | 1.55*** |
|  | (0.04),[<0.01] | (0.04),[<0.01] | (0.05),[<0.01] | (0.05),[<0.01] | (0.05),[<0.01] | (0.05),[<0.01] |
| Length of stay |  |  | 1.29*** | 1.41*** | 1.29*** | 1.41*** |
|  |  |  | (0.01),[<0.01] | (0.01),[<0.01] | (0.01),[<0.01] | (0.01),[<0.01] |
| Infection |  |  | 1.24*** | 2.02*** | 1.26*** | 2.05*** |
|  |  |  | (0.06),[<0.01] | (0.14),[<0.01] | (0.07),[<0.01] | (0.14),[<0.01] |
| Heart disease |  |  | 0.83*** | 0.77*** | 0.83*** | 0.76*** |
|  |  |  | (0.04),[<0.01] | (0.05),[<0.01] | (0.04),[<0.01] | (0.05),[<0.01] |
| Cerebrovascular |  |  | 2.02*** | 5.18*** | 2.07*** | 5.32*** |
|  |  |  | (0.13),[<0.01] | (0.38),[<0.01] | (0.13),[<0.01] | (0.40),[<0.01] |
| Geriatric |  |  | 1.69*** | 3.63*** | 1.69*** | 3.63*** |
|  |  |  | (0.10),[<0.01] | (0.27),[<0.01] | (0.10),[<0.01] | (0.27),[<0.01] |
| Trauma |  |  | 4.16*** | 20.27*** | 4.27*** | 20.82*** |
|  |  |  | (0.23),[<0.01] | (1.35),[<0.01] | (0.24),[<0.01] | (1.39),[<0.01] |
| Prior admission |  |  | 1.01 | 1.01 | 1.01 | 1.02 |
|  |  |  | (0.04),[0.90] | (0.05),[0.79] | (0.04),[0.84] | (0.05),[0.73] |
| Low income |  |  |  |  | 1.11** | 1.26*** |
|  |  |  |  |  | (0.05),[0.04] | (0.07),[<0.01] |
| Low education |  |  |  |  | 1.15*** | 1.16*** |
|  |  |  |  |  | (0.04),[<0.01] | (0.05),[<0.01] |
| Live alone |  |  |  |  | 1.60*** | 1.58*** |
|  |  |  |  |  | (0.05),[<0.01] | (0.06),[<0.01] |
| Num.Obs. | 44139 |  | 44139 |  | 44139 |  |
| AIC | 69640.1 |  | 58011.5 |  | 57626.5 |  |
| BIC | 69744.4 |  | 58237.6 |  | 57904.7 |  |
| LRT (Chi-square, p-value) |  |  | 11656 (p<.01) |  | 397 (p<.01) |  |

* p < 0.1, ** p < 0.05, *** p < 0.01. OR = Odds ratio, SE = Standard error, LRT = Likelihood ratio test. Model 1: model adjusted for age, sex and comorbidity. Model 2: Model 1 plus ICD-10 diagnostic groups, prior admission within 6 months and lengths of stay (LOS). Model 3: Model 2 plus income, education and living situation. Estimates for prior admission within 6 months and LOS are not shown.

Table S3.2: Odds ratios (OR), standard errors (SE), and p-values of *transitions into FCS (HHC or IC) at 6 months*, three different models

|  | Model 1 | | Model 2 | | Model 3 | |
| --- | --- | --- | --- | --- | --- | --- |
|  | To HHC | To IC | To HHC | To IC | To HHC | To IC |
| (Intercept) | 0.12*** | 0.11*** | 0.04*** | 0.02*** | 0.04*** | 0.01*** |
|  | (0.00),[<0.01] | (0.00),[<0.01] | (0.00),[<0.01] | (0.00),[<0.01] | (0.00),[<0.01] | (0.00),[<0.01] |
| Female | 1.44*** | 1.69*** | 1.46*** | 1.58*** | 1.27*** | 1.35*** |
|  | (0.04),[<0.01] | (0.05),[<0.01] | (0.04),[<0.01] | (0.05),[<0.01] | (0.04),[<0.01] | (0.04),[<0.01] |
| Age 80-84 | 1.69*** | 1.90*** | 1.75*** | 2.05*** | 1.70*** | 1.99*** |
|  | (0.06),[<0.01] | (0.06),[<0.01] | (0.06),[<0.01] | (0.08),[<0.01] | (0.06),[<0.01] | (0.07),[<0.01] |
| Age 85-89 | 2.83*** | 3.26*** | 3.05*** | 3.85*** | 2.84*** | 3.54*** |
|  | (0.10),[<0.01] | (0.12),[<0.01] | (0.12),[<0.01] | (0.16),[<0.01] | (0.11),[<0.01] | (0.15),[<0.01] |
| Age 90 + | 4.92*** | 7.36*** | 5.62*** | 9.74*** | 4.95*** | 8.41*** |
|  | (0.25),[<0.01] | (0.35),[<0.01] | (0.30),[<0.01] | (0.53),[<0.01] | (0.27),[<0.01] | (0.47),[<0.01] |
| Comorbid | 1.56*** | 1.59*** | 1.60*** | 1.94*** | 1.61*** | 1.96*** |
|  | (0.04),[<0.01] | (0.05),[<0.01] | (0.05),[<0.01] | (0.06),[<0.01] | (0.05),[<0.01] | (0.07),[<0.01] |
| Length of stay |  |  | 1.23*** | 1.34*** | 1.23*** | 1.34*** |
|  |  |  | (0.01),[<0.01] | (0.01),[<0.01] | (0.01),[<0.01] | (0.01),[<0.01] |
| Infection |  |  | 1.22*** | 1.46*** | 1.24*** | 1.49*** |
|  |  |  | (0.06),[<0.01] | (0.08),[<0.01] | (0.06),[<0.01] | (0.08),[<0.01] |
| Heart disease |  |  | 0.85*** | 0.64*** | 0.85*** | 0.64*** |
|  |  |  | (0.04),[<0.01] | (0.04),[<0.01] | (0.04),[<0.01] | (0.04),[<0.01] |
| Cerebrovascular |  |  | 1.92*** | 3.53*** | 1.97*** | 3.62*** |
|  |  |  | (0.12),[<0.01] | (0.23),[<0.01] | (0.12),[<0.01] | (0.24),[<0.01] |
| Geriatric |  |  | 1.39*** | 2.46*** | 1.39*** | 2.46*** |
|  |  |  | (0.08),[<0.01] | (0.15),[<0.01] | (0.08),[<0.01] | (0.16),[<0.01] |
| Trauma |  |  | 3.37*** | 11.03*** | 3.45*** | 11.31*** |
|  |  |  | (0.18),[<0.01] | (0.62),[<0.01] | (0.19),[<0.01] | (0.64),[<0.01] |
| Prior admission |  |  | 1.10** | 1.21*** | 1.10** | 1.22*** |
|  |  |  | (0.05),[0.03] | (0.06),[<0.01] | (0.05),[0.02] | (0.06),[<0.01] |
| Low income |  |  |  |  | 1.15*** | 1.29*** |
|  |  |  |  |  | (0.06),[<0.01] | (0.06),[<0.01] |
| Low education |  |  |  |  | 1.18*** | 1.18*** |
|  |  |  |  |  | (0.04),[<0.01] | (0.04),[<0.01] |
| Live alone |  |  |  |  | 1.50*** | 1.53*** |
|  |  |  |  |  | (0.05),[<0.01] | (0.05),[<0.01] |
| Num.Obs. | 41487 |  | 41487 |  | 41487 |  |
| AIC | 71554.9 |  | 62667.2 |  | 62300.4 |  |
| BIC | 71658.5 |  | 62891.7 |  | 62576.6 |  |
| LRT (Chi-square, p-value) |  |  | 8915 (p< 0.01) | | 378 (p < 0.01) |  |

* p < 0.1, ** p < 0.05, *** p < 0.01. OR = Odds ratio, SE = standard error. Model 1: model adjusted for age, sex and comorbidity. Model 2: Model 1 plus ICD-10 diagnostic groups, prior admission within 6 months and lengths of stay (LOS). Model 3: Model 2 plus income, education and living situation. Estimates for prior admission within 6 months and LOS are not shown.

Table S4.1 Odds ratios (OR), standard errors (SE), and p-values of *transitions into IC within 4 weeks*, three different models.

|  | Model 1 | Model 2 | Model 3 |
| --- | --- | --- | --- |
| (Intercept) | 0.35*** | 0.08*** | 0.07*** |
|  | (0.02),[<0.01] | (0.01),[<0.01] | (0.01),[<0.01] |
| Female | 1.18*** | 1.13*** | 1.11*** |
|  | (0.03),[<0.01] | (0.03),[<0.01] | (0.03),[<0.01] |
| Age 80-84 | 1.26*** | 1.29*** | 1.28*** |
|  | (0.06),[<0.01] | (0.06),[<0.01] | (0.06),[<0.01] |
| Age 85-89 | 1.52*** | 1.60*** | 1.59*** |
|  | (0.06),[<0.01] | (0.06),[<0.01] | (0.06),[<0.01] |
| Age 90 + | 1.85*** | 2.06*** | 2.02*** |
|  | (0.07),[<0.01] | (0.08),[<0.01] | (0.08),[<0.01] |
| Poor functional status | 3.02*** | 2.89*** | 2.90*** |
|  | (0.12),[<0.01] | (0.13),[<0.01] | (0.13),[<0.01] |
| Length of stay |  | 1.22*** | 1.22*** |
|  |  | (0.01),[<0.01] | (0.01),[<0.01] |
| Infection |  | 1.67*** | 1.68*** |
|  |  | (0.10),[<0.01] | (0.10),[<0.01] |
| Heart disease |  | 1.14** | 1.14** |
|  |  | (0.07),[0.02] | (0.07),[0.03] |
| Cerebrovascular |  | 2.42*** | 2.42*** |
|  |  | (0.16),[<0.01] | (0.15),[<0.01] |
| Geriatric |  | 2.43*** | 2.43*** |
|  |  | (0.12),[<0.01] | (0.12),[<0.01] |
| Trauma |  | 6.61*** | 6.60*** |
|  |  | (0.47),[<0.01] | (0.47),[<0.01] |
| Prior admission |  | 1.06 | 1.06* |
|  |  | (0.04),[0.10] | (0.04),[0.09] |
| Low income |  |  | 1.00 |
|  |  |  | (0.04),[0.97] |
| Low education |  |  | 1.00 |
|  |  |  | (0.04),[0.91] |
| Live alone |  |  | 1.09*** |
|  |  |  | (0.03),[<0.01] |
| Num.Obs. | 37536 | 37536 | 37536 |
| AIC | 48418.7 | 43299.7 | 43294.4 |
| BIC | 48469.9 | 43410.6 | 43430.9 |
| Log.Lik. | -24203.353 | -21636.825 | -21631.201 |
| LRT (Chi-square, p-value) |  | 5133 (p<0.01) | 11 (p < 0.05) |

* p < 0.1, ** p < 0.05, *** p < 0.01. OR = Odds ratio, SE = standard error, Model 1: model adjusted for age, sex and comorbidity. Model 2: Model 1 plus ICD-10 diagnostic groups, prior admission within 6 months and lengths of stay (LOS). Model 3: Model 2 plus income, education and living situation. Estimates for prior admission within 6 months and LOS are not shown.

Table S4.2 Odds ratios (OR), standard errors (SE), and p-values of *transitions into IC at 6 months*, three different models.

|  | Model 1 | Model 2 | Model 3 |
| --- | --- | --- | --- |
| (Intercept) | 0.56*** | 0.19*** | 0.18*** |
|  | (0.03),[<0.01] | (0.02),[<0.01] | (0.02),[<0.01] |
| Female | 1.17*** | 1.14*** | 1.13*** |
|  | (0.04),[<0.01] | (0.04),[<0.01] | (0.04),[<0.01] |
| Age 80-84 | 1.17*** | 1.19*** | 1.19*** |
|  | (0.05),[<0.01] | (0.06),[<0.01] | (0.06),[<0.01] |
| Age 85-89 | 1.46*** | 1.53*** | 1.53*** |
|  | (0.06),[<0.01] | (0.07),[<0.01] | (0.07),[<0.01] |
| Age 90 + | 1.76*** | 1.92*** | 1.91*** |
|  | (0.08),[<0.01] | (0.10),[<0.01] | (0.10),[<0.01] |
| Poor functional status | 3.73*** | 3.51*** | 3.52*** |
|  | (0.19),[<0.01] | (0.18),[<0.01] | (0.18),[<0.01] |
| Length of stay |  | 1.16*** | 1.16*** |
|  |  | (0.01),[<0.01] | (0.01),[<0.01] |
| Infection |  | 1.35*** | 1.35*** |
|  |  | (0.07),[<0.01] | (0.07),[<0.01] |
| Heart disease |  | 0.94 | 0.94 |
|  |  | (0.05),[0.28] | (0.05),[0.28] |
| Cerebrovascular |  | 1.67*** | 1.67*** |
|  |  | (0.11),[<0.01] | (0.11),[<0.01] |
| Geriatric |  | 2.03*** | 2.03*** |
|  |  | (0.10),[<0.01] | (0.10),[<0.01] |
| Trauma |  | 4.55*** | 4.55*** |
|  |  | (0.31),[<0.01] | (0.31),[<0.01] |
| Prior admission |  | 1.35*** | 1.35*** |
|  |  | (0.05),[<0.01] | (0.05),[<0.01] |
| Low income |  |  | 0.95 |
|  |  |  | (0.04),[0.27] |
| Low education |  |  | 1.05 |
|  |  |  | (0.05),[0.31] |
| Live alone |  |  | 1.06* |
|  |  |  | (0.04),[0.07] |
| Num.Obs. | 30528 | 30528 | 30528 |
| AIC | 39294.0 | 36480.7 | 36480.0 |
| BIC | 39343.9 | 36588.9 | 36613.2 |
| LRT (Chi-square, p-value) |  | 2827 (p < 0.01) | 6 (p = 0.08) |

* p < 0.1, ** p < 0.05, *** p < 0.01. OR = Odds ratio, SE = standard error, Model 1: model adjusted for age, sex and comorbidity. Model 2: Model 1 plus ICD-10 diagnostic groups, prior admission within 6 months and lengths of stay (LOS). Model 3: Model 2 plus income, education and living situation. Estimates for prior admission within 6 months and LOS are not shown.

Table S5: Odds ratios (OR), standard errors (SE), and p-values *of transitions into FCS (HHC or IC)* within 4 weeks and at 6 months for a full model with interactions.

|  | 4 weeks | | 6 months | |
| --- | --- | --- | --- | --- |
|  | To HHC | To IC | To HHC | To IC |
| (Intercept) | 0.02*** | 0.01*** | 0.04*** | 0.01*** |
|  | (0.00),[<0.01] | (0.00),[<0.01] | (0.00),[<0.01] | (0.00),[<0.01] |
| Female | 1.06 | 1.07 | 1.14* | 1.01 |
|  | (0.09),[0.52] | (0.13),[0.58] | (0.09),[0.08] | (0.09),[0.94] |
| Age 80-84 | 1.60*** | 1.81*** | 1.70*** | 1.99*** |
|  | (0.06),[<0.01] | (0.07),[<0.01] | (0.06),[<0.01] | (0.07),[<0.01] |
| Age 85-89 | 2.66*** | 3.45*** | 2.83*** | 3.53*** |
|  | (0.11),[<0.01] | (0.15),[<0.01] | (0.11),[<0.01] | (0.15),[<0.01] |
| Age 90 + | 4.51*** | 7.73*** | 4.94*** | 8.42*** |
|  | (0.24),[<0.01] | (0.43),[<0.01] | (0.27),[<0.01] | (0.47),[<0.01] |
| Comorbid | 1.73*** | 1.76*** | 1.69*** | 2.32*** |
|  | (0.14),[<0.01] | (0.21),[<0.01] | (0.13),[<0.01] | (0.21),[<0.01] |
| Length of stay | 1.28*** | 1.41*** | 1.22*** | 1.34*** |
|  | (0.01),[<0.01] | (0.01),[<0.01] | (0.01),[<0.01] | (0.01),[<0.01] |
| Infection | 1.45*** | 1.91*** | 1.42*** | 1.47*** |
|  | (0.14),[<0.01] | (0.24),[<0.01] | (0.12),[<0.01] | (0.15),[<0.01] |
| Heart disease | 0.74*** | 0.60*** | 0.74*** | 0.51*** |
|  | (0.07),[<0.01] | (0.08),[<0.01] | (0.06),[<0.01] | (0.05),[<0.01] |
| Cerebrovascular | 2.05*** | 4.27*** | 1.83*** | 3.11*** |
|  | (0.22),[<0.01] | (0.56),[<0.01] | (0.19),[<0.01] | (0.35),[<0.01] |
| Geriatric | 1.94*** | 4.08*** | 1.55*** | 2.71*** |
|  | (0.22),[<0.01] | (0.58),[<0.01] | (0.17),[<0.01] | (0.32),[<0.01] |
| Trauma | 3.40*** | 14.92*** | 2.68*** | 8.46*** |
|  | (0.36),[<0.01] | (1.86),[<0.01] | (0.27),[<0.01] | (0.89),[<0.01] |
| Prior admission | 1.00 | 1.01 | 1.09** | 1.20*** |
|  | (0.04),[0.97] | (0.05),[0.87] | (0.05),[0.04] | (0.06),[<0.01] |
| Low income | 1.11** | 1.27*** | 1.16*** | 1.29*** |
|  | (0.05),[0.03] | (0.07),[<0.01] | (0.06),[<0.01] | (0.06),[<0.01] |
| Low education | 1.15*** | 1.16*** | 1.18*** | 1.17*** |
|  | (0.04),[<0.01] | (0.05),[<0.01] | (0.04),[<0.01] | (0.04),[<0.01] |
| Live alone | 1.69*** | 1.36** | 1.53*** | 1.49*** |
|  | (0.14),[<0.01] | (0.17),[0.01] | (0.12),[<0.01] | (0.14),[<0.01] |
| Infection:Female | 1.02 | 1.11 | 1.00 | 1.23* |
|  | (0.11),[0.83] | (0.16),[0.45] | (0.10),[0.97] | (0.14),[0.07] |
| Heart disease:Female | 1.30*** | 1.34** | 1.20* | 1.40*** |
|  | (0.13),[<0.01] | (0.19),[0.04] | (0.11),[0.05] | (0.16),[<0.01] |
| Cerebrovascular:Female | 1.38** | 1.59*** | 1.19 | 1.69*** |
|  | (0.18),[0.01] | (0.25),[<0.01] | (0.15),[0.17] | (0.23),[<0.01] |
| Geriatric:Female | 0.80* | 0.96 | 0.78** | 1.09 |
|  | (0.10),[0.09] | (0.15),[0.79] | (0.10),[0.04] | (0.15),[0.50] |
| Trauma:Female | 1.83*** | 1.65*** | 1.67*** | 1.77*** |
|  | (0.22),[<0.01] | (0.23),[<0.01] | (0.19),[<0.01] | (0.21),[<0.01] |
| Infection:Comorbid | 0.76*** | 0.76** | 0.82** | 0.73*** |
|  | (0.08),[<0.01] | (0.10),[0.04] | (0.08),[0.05] | (0.08),[<0.01] |
| Heart disease:Comorbid | 0.94 | 1.01 | 1.08 | 1.04 |
|  | (0.09),[0.56] | (0.14),[0.95] | (0.10),[0.41] | (0.12),[0.72] |
| Cerebrovascular:Comorbid | 0.68*** | 0.93 | 0.85 | 0.81 |
|  | (0.10),[<0.01] | (0.15),[0.64] | (0.12),[0.24] | (0.12),[0.15] |
| Geriatric:Comorbid | 0.74** | 0.84 | 0.87 | 0.78* |
|  | (0.10),[0.02] | (0.14),[0.29] | (0.11),[0.30] | (0.11),[0.07] |
| Trauma:Comorbid | 0.75** | 0.77* | 0.83 | 0.67*** |
|  | (0.09),[0.02] | (0.11),[0.07] | (0.10),[0.15] | (0.08),[<0.01] |
| Infection:Live alone | 0.86 | 1.27* | 0.83* | 1.05 |
|  | (0.09),[0.15] | (0.18),[0.09] | (0.08),[0.07] | (0.12),[0.69] |
| Heart disease:Live alone | 0.98 | 1.16 | 1.01 | 1.02 |
|  | (0.10),[0.82] | (0.17),[0.31] | (0.09),[0.88] | (0.12),[0.88] |
| Cerebrovascular:Live alone | 0.90 | 0.98 | 1.06 | 0.88 |
|  | (0.12),[0.45] | (0.15),[0.92] | (0.14),[0.63] | (0.12),[0.36] |
| Geriatric:Live alone | 1.19 | 0.95 | 1.17 | 0.90 |
|  | (0.15),[0.18] | (0.15),[0.75] | (0.14),[0.20] | (0.12),[0.41] |
| Trauma:Live alone | 0.91 | 1.30* | 0.98 | 1.20 |
|  | (0.11),[0.41] | (0.18),[0.06] | (0.11),[0.86] | (0.14),[0.12] |
| Num.Obs. | 44139 |  | 41487 |  |
| AIC | 57555.4 |  | 62228.5 |  |
| BIC | 58094.5 |  | 62763.8 |  |

* p < 0.1, ** p < 0.05, *** p < 0.01. Estimates are based on a full model (Model 3) plus interaction term between diagnostic group and selected patient characteristics (sex, comorbidity and living situation). Estimates for prior admission within 6 months and LOS are not shown.

Table S6: Odds ratios (OR), standard errors (SE), and p-values of *transitions into IC* within 4 weeks and at 6 months for a full model with interactions.

|  | 4 weeks | 6 months |
| --- | --- | --- |
| (Intercept) | 0.08*** | 0.20*** |
|  | (0.01),[<0.01] | (0.02),[<0.01] |
| Female | 0.86* | 0.97 |
|  | (0.07),[0.06] | (0.09),[0.73] |
| Age 80-84 | 1.29*** | 1.19*** |
|  | (0.06),[<0.01] | (0.06),[<0.01] |
| Age 85-89 | 1.60*** | 1.54*** |
|  | (0.06),[<0.01] | (0.07),[<0.01] |
| Age 90 + | 2.03*** | 1.92*** |
|  | (0.08),[<0.01] | (0.10),[<0.01] |
| Poor functional status | 3.37*** | 3.63*** |
|  | (0.25),[<0.01] | (0.36),[<0.01] |
| Length of stay | 1.22*** | 1.16*** |
|  | (0.01),[<0.01] | (0.01),[<0.01] |
| Infection | 1.54*** | 1.18** |
|  | (0.14),[<0.01] | (0.09),[0.04] |
| Heart disease | 0.89 | 0.76*** |
|  | (0.09),[0.25] | (0.07),[<0.01] |
| Cerebrovascular | 1.83*** | 1.23* |
|  | (0.21),[<0.01] | (0.15),[0.09] |
| Geriatric | 2.21*** | 1.90*** |
|  | (0.21),[<0.01] | (0.16),[<0.01] |
| Trauma | 6.82*** | 4.50*** |
|  | (0.79),[<0.01] | (0.57),[<0.01] |
| Prior admission | 1.06 | 1.35*** |
|  | (0.04),[0.10] | (0.05),[<0.01] |
| Low income | 1.00 | 0.95 |
|  | (0.04),[0.96] | (0.05),[0.27] |
| Low education | 1.01 | 1.05 |
|  | (0.04),[0.85] | (0.05),[0.28] |
| Live alone | 1.08 | 1.01 |
|  | (0.09),[0.36] | (0.08),[0.92] |
| Infection:Female | 1.32*** | 1.24** |
|  | (0.13),[<0.01] | (0.13),[0.04] |
| Heart disease:Female | 1.42*** | 1.23* |
|  | (0.14),[<0.01] | (0.14),[0.06] |
| Cerebrovascular:Female | 1.16 | 1.13 |
|  | (0.16),[0.25] | (0.18),[0.43] |
| Geriatric:Female | 1.24*** | 1.03 |
|  | (0.09),[<0.01] | (0.10),[0.76] |
| Trauma:Female | 1.33*** | 1.22* |
|  | (0.13),[<0.01] | (0.14),[0.08] |
| Infection:Poor functional status | 0.80** | 0.87 |
|  | (0.08),[0.02] | (0.11),[0.27] |
| Heart disease:Poor functional status | 1.03 | 1.17 |
|  | (0.09),[0.76] | (0.14),[0.18] |
| Cerebrovascular:Poor functional status | 1.36** | 1.63*** |
|  | (0.18),[0.02] | (0.28),[<0.01] |
| Geriatric:Poor functional status | 0.94 | 1.17 |
|  | (0.08),[0.44] | (0.14),[0.19] |
| Trauma:Poor functional status | 0.52*** | 0.60*** |
|  | (0.06),[<0.01] | (0.09),[<0.01] |
| Infection:Live alone | 0.99 | 1.09 |
|  | (0.09),[0.91] | (0.09),[0.32] |
| Heart disease:Live alone | 1.06 | 1.10 |
|  | (0.13),[0.61] | (0.10),[0.31] |
| Cerebrovascular:Live alone | 1.14 | 1.19 |
|  | (0.17),[0.38] | (0.19),[0.26] |
| Geriatric:Live alone | 0.97 | 1.00 |
|  | (0.11),[0.78] | (0.10),[0.99] |
| Trauma:Live alone | 0.97 | 0.99 |
|  | (0.11),[0.79] | (0.10),[0.91] |
| Num.Obs. | 37536 | 30528 |
| AIC | 43213.1 | 36427.0 |
| BIC | 43477.6 | 36685.1 |

* p < 0.1, ** p < 0.05, *** p < 0.01. Estimates are based on a full model (Model 3) plus interaction term between diagnostic group and selected patient characteristics (sex, functional status and living situation). Estimates for prior admission within 6 months and LOS are not shown.
